# Viral epitope profiling of COVID-19 patients reveals cross-reactivity and correlates of severity

**DOI:** 10.1126/science.abd4250

**Published:** 2020-09-29

**Authors:** Ellen Shrock, Eric Fujimura, Tomasz Kula, Richard T. Timms, I-Hsiu Lee, Yumei Leng, Matthew L. Robinson, Brandon M. Sie, Mamie Z. Li, Yuezhou Chen, Jennifer Logue, Adam Zuiani, Denise McCulloch, Felipe J. N. Lelis, Stephanie Henson, Daniel R. Monaco, Meghan Travers, Shaghayegh Habibi, William A. Clarke, Patrizio Caturegli, Oliver Laeyendecker, Alicja Piechocka-Trocha, Jonathan Z. Li, Ashok Khatri, Helen Y. Chu, MGH COVID-19 Collection & Processing Team, Alexandra-Chloé Villani, Kyle Kays, Marcia B. Goldberg, Nir Hacohen, Michael R. Filbin, Xu G. Yu, Bruce D. Walker, Duane R. Wesemann, H. Benjamin Larman, James A. Lederer, Stephen J. Elledge

**Affiliations:** 1 Department of Genetics, Harvard Medical School, Boston, MA, USA.; 2 Howard Hughes Medical Institute, Division of Genetics, Brigham and Women’s Hospital, Program in Virology, Harvard Medical School, Boston, MA, USA.; 3 Chemical Biology Program, Harvard University, Cambridge, MA, USA.; 4 Center for Systems Biology, Department of Radiology, Massachusetts General Hospital and Harvard Medical School, Boston, MA, USA.; 5 Division of Infectious Diseases, Department of Medicine, Johns Hopkins University School of Medicine, Baltimore, MD, USA.; 6 Division of Allergy and Immunology and Division of Genetics, Department of Medicine, Brigham and Women’s Hospital, Harvard Medical School, Boston, MA, USA.; 7 Massachusetts Consortium on Pathogen Readiness, Boston, MA, USA.; 8 Department of Medicine, University of Washington, Seattle, WA, USA.; 9 Institute for Cell Engineering, Immunology Division, Department of Pathology, Johns Hopkins University, Baltimore, MD, USA.; 10 Division of Clinical Chemistry, Department of Pathology, Johns Hopkins School of Medicine, Baltimore, MD, USA.; 11 Division of Immunology, Department of Pathology, Johns Hopkins School of Medicine, Baltimore, MD, USA.; 12 Division of Intramural Research, NIAID, NIH, Baltimore, MD, USA.; 13 Howard Hughes Medical Institute, Ragon Institute of MGH, MIT and Harvard, Cambridge, MA, USA.; 14 Infectious Disease Division, Department of Medicine, Brigham and Women’s Hospital, Boston, MA, USA.; 15 Endocrine Unit and Department of Medicine, Massachusetts General Hospital, Harvard Medical School, Boston, MA, USA.; 16 Massachusetts General Hospital, Harvard Medical School, Boston, MA 02115, USA.; 17 Massachusetts General Hospital Center for Immunology and Inflammatory Diseases, Massachusetts General Hospital Cancer Center, Department of Medicine, Harvard Medical School, Boston, MA, USA.; 18 Department of Emergency Medicine, Massachusetts General Hospital, Boston, MA, USA.; 19 Center for Bacterial Pathogenesis, Division of Infectious Diseases, Department of Medicine and Microbiology, Massachusetts General Hospital and Harvard Medical School, Boston, MA, USA.; 20 Massachusetts General Hospital Cancer Center, Department of Medicine, Harvard Medical School, Boston, MA, USA.; 21 Ragon Institute of MGH, MIT and Harvard, Cambridge, MA, USA.; 22 Centre for the AIDS Programme of Research in South Africa, Congella, South Africa.; 23 Department of Surgery, Brigham and Women's Hospital and Harvard Medical School, Boston, MA, USA.

## Abstract

Understanding humoral responses to severe acute respiratory syndrome coronavirus 2 (SARS-CoV-2) is critical for improving diagnostics, therapeutics, and vaccines. Deep serological profiling of 232 coronavirus disease 2019 (COVID-19) patients and 190 pre–COVID-19 era controls using VirScan revealed more than 800 epitopes in the SARS-CoV-2 proteome, including 10 epitopes likely recognized by neutralizing antibodies. Preexisting antibodies in controls recognized SARS-CoV-2 ORF1, whereas only COVID-19 patient antibodies primarily recognized spike protein and nucleoprotein. A machine learning model trained on VirScan data predicted SARS-CoV-2 exposure history with 99% sensitivity and 98% specificity; a rapid Luminex-based diagnostic was developed from the most discriminatory SARS-CoV-2 peptides. Individuals with more severe COVID-19 exhibited stronger and broader SARS-CoV-2 responses, weaker antibody responses to prior infections, and higher incidence of cytomegalovirus and herpes simplex virus 1, possibly influenced by demographic covariates. Among hospitalized patients, males produce stronger SARS-CoV-2 antibody responses than females.

## Profiling coronaviruses

Among the coronaviruses that infect humans, four cause mild common colds, whereas three others, including the currently circulating severe acute respiratory syndrome coronavirus 2 (SARS-CoV-2), result in severe infections. Shrock *et al.* used a technology known as VirScan to probe the antibody repertoires of hundreds of coronavirus disease 2019 (COVID-19) patients and pre–COVID-19 era controls. They identified hundreds of antibody targets, including several antibody epitopes shared by the mild and severe coronaviruses and many specific to SARS-CoV-2. A machine-learning model accurately classified patients infected with SARS-CoV-2 and guided the design of an assay for rapid SARS-CoV-2 antibody detection. The study also looked at how the antibody response and viral exposure history differ in patients with diverging outcomes, which could inform the production of improved vaccine and antibody therapies.

*Science*, this issue p. eabd4250

## Abstract (structured)

**INTRODUCTION:**

A systematic characterization of the humoral response to severe acute respiratory system coronavirus 2 (SARS-CoV-2) epitopes has yet to be performed. This analysis is important for understanding the immunogenicity of the viral proteome and the basis for cross-reactivity with the common-cold coronaviruses.

Coronavirus disease 2019 (COVID-19), caused by SARS-CoV-2, is notable for its variable course, with some individuals remaining asymptomatic whereas others experience fever, respiratory distress, or even death. A comprehensive investigation of the antibody response in individuals with severe versus mild COVID-19—as well as an examination of past viral exposure history—is needed.

**RATIONALE:**

An understanding of humoral responses to SARS-CoV-2 is critical for improving diagnostics and vaccines and gaining insight into variable clinical outcomes. To this end, we used VirScan, a high-throughput method to analyze epitopes of antiviral antibodies in human sera. We supplemented the original VirScan library with additional libraries of peptides spanning the proteomes of SARS-CoV-2 and all other human coronaviruses. These libraries enabled us to precisely map epitope locations and investigate cross-reactivity between SARS-CoV-2 and other coronavirus strains. The original VirScan library allowed us to simultaneously investigate antibody responses to prior infections and viral exposure history.

**RESULTS:**

We screened sera from 232 COVID-19 patients and 190 pre–COVID-19 era controls against the original VirScan and supplemental coronavirus libraries, assaying more than 10^8^ antibody repertoire–peptide interactions. We identified epitopes ranging from “private” (recognized by antibodies in only a small number of individuals) to “public” (recognized by antibodies in many individuals) and detected SARS-CoV-2–specific epitopes as well as those that cross-react with common-cold coronaviruses. Several of these cross-reacting antibodies are present in pre–COVID-19 era samples. We developed a machine learning model that predicted SARS-CoV-2 exposure history with 99% sensitivity and 98% specificity from VirScan data. We used the most discriminatory SARS-CoV-2 peptides to produce a Luminex-based serological assay, which performed similarly to gold-standard enzyme-linked immunosorbent assays. We stratified the COVID-19 patient samples by disease severity and found that patients who had required hospitalization exhibited stronger and broader antibody responses to SARS-CoV-2 but weaker overall responses to past infections compared with those who did not need hospitalization. Further, the hospitalized group had higher seroprevalence rates for cytomegalovirus and herpes simplex virus 1. These findings may be influenced by differences in demographic compositions between the two groups, but they raise hypotheses that may be tested in future studies. Using alanine scanning mutagenesis, we precisely mapped 823 distinct epitopes across the entire SARS-CoV-2 proteome, 10 of which are likely targets of neutralizing antibodies. One cross-reactive antibody epitope in S2 has been previously suggested to be neutralizing and, as it exists in pre–COVID-19 era samples, could affect the severity of COVID-19.

**CONCLUSION:**

We present a highly detailed view of the epitope landscape within the SARS-CoV-2 proteome. This knowledge may be used to produce diagnostics with improved specificity and can provide a stepping stone to the isolation and functional dissection of both neutralizing antibodies and antibodies that might exacerbate patient outcomes through antibody-dependent enhancement or immune distraction.

Our study reveals notable correlations between COVID-19 severity and both viral exposure history and overall strength of the antibody response to past infections. These findings are likely influenced by demographic covariates, but they generate hypotheses that may be tested with larger patient cohorts matched for age, gender, race, and other demographic variables.

## Teaser

Deep serological profiling reveals discriminatory epitopes that could be used for SARS-CoV-2 antibody detection.

## Graphical abstract

**Figure:**
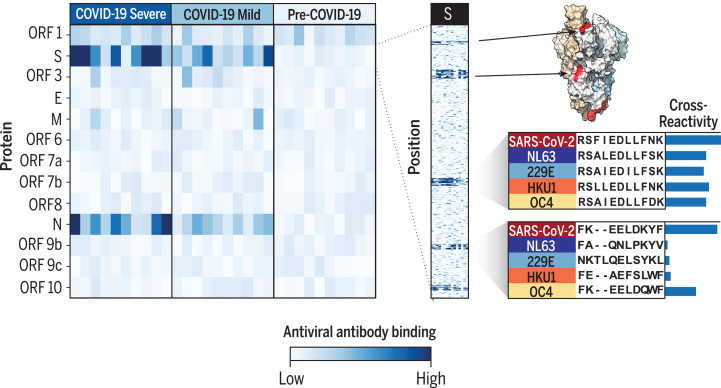
SARS-CoV-2 epitope mapping. VirScan detects antibodies against SARS-CoV-2 in COVID-19 patients with severe and mild disease. Heatmap color represents the strength of the antibody response in each sample (columns) to each protein (rows, left) or peptide (rows, right). VirScan reveals the precise positions of epitopes, which can be mapped onto the structure of the spike protein (S). Examination of SARS-CoV-2 and seasonal coronavirus sequence conservation explains epitope cross-reactivity. A, Ala; D, Asp; E, Glu; F, Phe; I, Ile; K, Lys; L, Leu; N, Asn; P, Pro; Q, Gln; R, Arg; S, Ser; T, Thr; V, Val; W, Trp; Y, Tyr.

Cornaviruses constitute a large family of enveloped, positive-sense single-stranded RNA viruses that cause diseases in birds and mammals (*1*). Among the strains that infect humans are the alphacoronaviruses HCoV-229E and HCoV-NL63 and the betacoronaviruses HCoV-OC43 and HCoV-HKU1, which cause common colds (Fig. 1A). Three additional betacoronavirus species result in severe infections in humans: severe acute respiratory syndrome coronavirus (SARS-CoV), Middle East respiratory syndrome coronavirus (MERS-CoV), and severe acute respiratory syndrome coronavirus 2 (SARS-CoV-2), a novel coronavirus that emerged in late 2019 in Asia and quickly spread throughout the world (*2*). As of November 2020, SARS-CoV-2 has caused more than 50 million confirmed infections and nearly 1.3 million deaths (*3*).

**Fig. 1:**
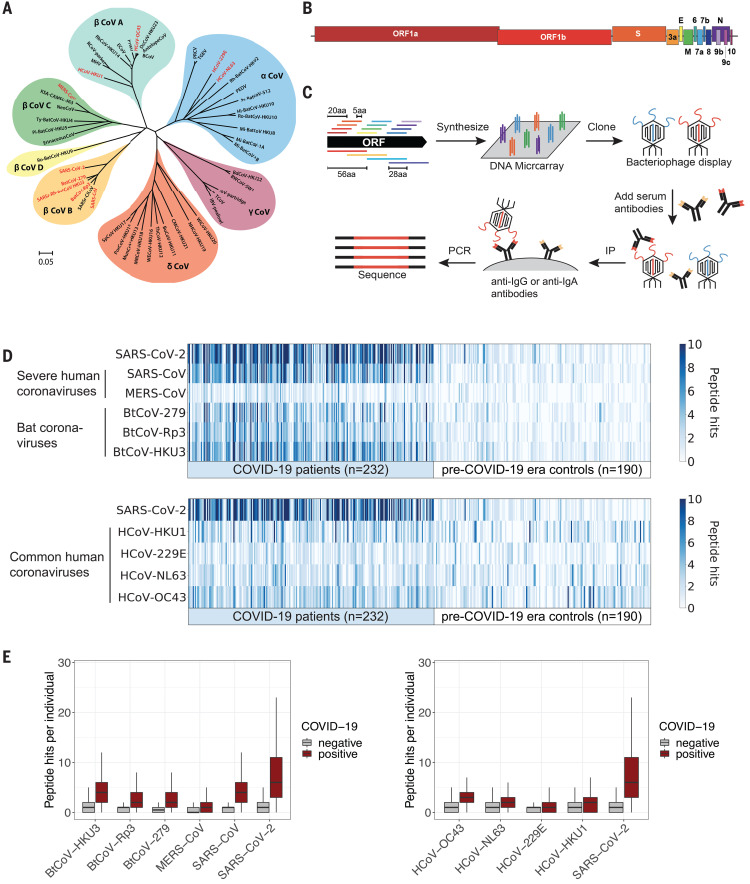
VirScan detects the humoral response to SARS-CoV-2 in sera from COVID-19 patients. (**A**) Phylogeny tree of 50 coronavirus sequences (*32*) constructed using MEGA X (*33*, *34*). The scale bar indicates the estimated number of base substitutions per site (*35*). Coronaviruses included in the updated VirScan library are indicated in red. (**B**) Schematic representation of the ORFs encoded by the SARS-CoV-2 genome (*10*, *36*). (**C**) Overview of the VirScan procedure (*5*–*8*). The coronavirus oligonucleotide library includes 56-mer peptides tiling every 28 amino acids (aa) across the proteomes of 10 coronavirus strains and 20-mer peptides tiling every 5 amino acids across the SARS-CoV-2 proteome. Oligonucleotides were cloned into a T7 bacteriophage display vector and packaged into phage particles displaying the encoded peptides on their surface. The phage library was mixed with sera containing antibodies that bind to their cognate epitopes on the phage surface; bound phage were isolated by IP with either anti-IgG– or anti-IgA–coated magnetic beads. Lastly, PCR amplification and Illumina sequencing from the DNA of the bound phage revealed the peptides targeted by the serum antibodies. (**D**) Detection of antibodies targeting coronavirus epitopes by VirScan. Heatmaps depict the humoral response from COVID-19 patients (*n* = 232) and pre–COVID-19 era control samples (*n* = 190). Each column represents a sample from a distinct individual. The color intensity indicates the number of 56-mer peptides from the indicated coronaviruses significantly enriched by IgG antibodies in the serum sample. (**E**) Box plots illustrate the number of peptide hits from the indicated coronaviruses in COVID-19 patients and pre–COVID-19 era controls. The box indicates the interquartile range, with a line at the median. The whiskers span 1.5 times the interquartile range.

The clinical course of coronavirus disease 19 (COVID-19)—the disease resulting from SARS-CoV-2 infection—is notable for its extreme variability: Some individuals remain entirely asymptomatic, whereas others experience fever, anosmia, diarrhea, severe respiratory distress, pneumonia, cardiac arrhythmia, blood clotting disorders, liver and kidney distress, enhanced cytokine release and, in a small percentage of cases, death (*4*). Therefore, understanding the factors that influence this spectrum of outcomes is an intense area of research. Disease severity is correlated with advanced age, sex, ethnicity, socioeconomic status, and comorbidities such as diabetes, cardiovascular disease, chronic lung disease, obesity, and reduced immune function (*4*). Additional relevant factors likely include the inoculum of virus at infection and individual genetic background and viral exposure history. The complex interplay of these elements also determines how individuals respond to therapies aimed at mitigating disease severity. Detailed knowledge of the immune response to SARS-CoV-2 could improve our understanding of diverse outcomes and inform the development of improved diagnostics, vaccines, and antibody-based therapies.

In this study, we used VirScan, a programmable phage-display immunoprecipitation and sequencing (PhIP-Seq) technology that we developed previously, to explore antiviral antibody responses across the human virome (*5*–*8*). Here we describe a detailed analysis of the humoral response in COVID-19 patients.

## Results

### Development of a VirScan library targeting human coronaviruses

Our existing VirScan phage-display platform is based on an oligonucleotide library encoding 56–amino acid (56-mer) peptides tiling every 28 amino acids across the proteomes of all known pathogenic human viruses (~400 species and strains) plus many bacterial proteins (*8*). To investigate the serological response to SARS-CoV-2 and other human coronaviruses (HCoVs), we supplemented this library with three additional sublibraries: Sublibrary 1 encodes a 56-mer peptide library tiling every 28 amino acids through each of the open reading frames (ORFs) expressed by the six HCoVs and three bat coronaviruses closely related to SARS-CoV-2; sublibrary 2 encodes 20-mer peptides tiling every 5 amino acids across the SARS-CoV-2 proteome, enabling more precise localization of epitopes; and sublibrary 3 encodes triple-alanine scanning mutants of the 56-mer peptides tiling across the SARS-CoV-2 proteome, enabling the mapping of epitope boundaries at amino acid resolution (Fig. 1, A to C, and table S1) (*9*, *10*).

We used VirScan (Fig. 1C) to profile the antibody repertoires of nine cohorts of individuals from multiple locations, including Baltimore, MD, Boston, MA, and Seattle, WA (tables S2 to S8). These cohorts comprised longitudinal samples from individuals enrolled in prospective studies of COVID-19 infection, cross-sectional samples from patients with active COVID-19 who were receiving treatment in either hospital or outpatient settings, and cross-sectional samples from convalescent individuals with a past history of COVID-19. Our cohorts also included a diverse set of control sera collected before the COVID-19 outbreak. We profiled the targets of IgG and IgA (immunoglobulins G and A) antibodies separately: IgG and IgA are the most abundant isotypes in blood, whereas IgA is the principal isotype secreted on mucosal surfaces, including the respiratory tract. Collectively, we analyzed ~550 samples in duplicate, in total assessing ~100 million potential antibody repertoire–peptide interactions.

### Detection of SARS-CoV-2 seropositivity with VirScan

To measure immune responses to SARS-CoV-2, we compared VirScan profiles of serum samples from COVID-19 patients to those of controls obtained before the emergence of SARS-CoV-2 in 2019. These pre–COVID-19 era controls facilitate identification of (i) SARS-CoV-2 peptides encoding epitopes specific to COVID-19 patients and (ii) SARS-CoV-2 peptides encoding epitopes that are cross-reactive with antibodies developed in response to the ubiquitous common-cold HCoVs. Sera from COVID-19 patients exhibited much more SARS-CoV-2 reactivity than did sera from pre–COVID-19 era controls (Fig. 1, D and E). Some cross-reactivity toward SARS-CoV-2 peptides was observed in the pre–COVID-19 era samples, but this was expected because nearly all people have been exposed to one or more HCoVs (*11*).

COVID-19 patient sera also showed significant levels of cross-reactivity with the other highly pathogenic HCoVs, SARS-CoV and MERS-CoV, although less was observed against the more distantly related MERS-CoV. Extensive cross-reactivity was also observed against peptides derived from the three bat coronaviruses that share the greatest proportion of sequence identity with SARS-CoV-2 (Fig. 1, A, D, and E) (*9*). We know that these represent cross-reactivities because, given the low prevalence and circumscribed geographical location of SARS-CoV and MERS-CoV, none of the individuals in this study are likely to have encountered these viruses.

COVID-19 patient sera also exhibited a significantly higher level of reactivity to seasonal HCoV peptides than did sera from pre–COVID-19 era controls (Fig. 1, D and E). This could be due to the elicitation of novel antibodies that cross-react or to an anamnestic response that boosts B cell memory against HCoVs. The converse is not always true: Many pre–COVID-19 era samples exhibit strong recognition of seasonal HCoV peptides but little or no recognition of SARS-CoV-2 peptides (Fig. 1D). In some cases, the concentrations of antibodies against seasonal HCoVs may be below the threshold of detection in the pre–COVID-19 era samples.

### Coronavirus proteins targeted by antibodies in COVID-19 patients

**Fig. 2:**
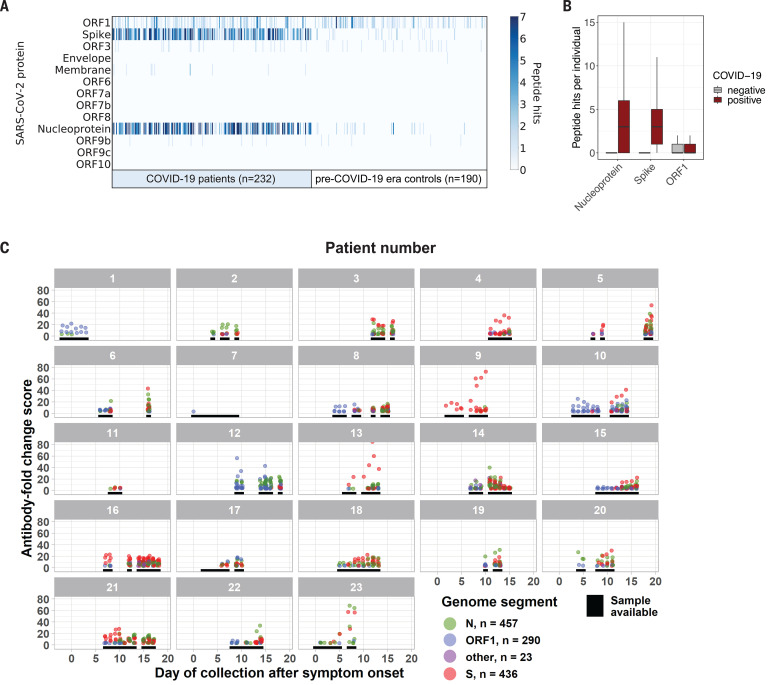
SARS-CoV-2 protein recognition in COVID-19 patient versus control sera. (**A**) Antibodies targeting SARS-CoV-2 proteins. Each column represents a distinct patient sample, and each row represents a SARS-CoV-2 protein. The color intensity in each cell of the heatmap indicates the number of 56-mer peptides, as in Fig. 1D. (**B**) Box plots (as in Fig. 1E) illustrate the number of peptide hits from each of the indicated SARS-CoV-2 proteins detected in the IgG antibody response of COVID-19 patients and controls. (**C**) Longitudinal analysis of the antibody response to SARS-CoV-2 for 23 patients with confirmed COVID-19. Black lines indicate days when a sample was available for analysis. Each point represents the maximum antibody fold-change score per SARS-CoV-2 peptide in each sample, colored by protein target.

Analysis of SARS-CoV-2 proteins targeted by COVID-19 patient antibodies revealed that the primary responses to SARS-CoV-2 are reactive with peptides derived from spike protein (S) and nucleoprotein (N) (Fig. 2, A and B). Compared with sera from pre–COVID-19 era controls, COVID-19 patient sera exhibit significant differential recognition of these two proteins, indicating that this recognition is a result of antibody responses to SARS-CoV-2. Third-most frequently recognized is the replicase polyprotein ORF1, but unlike S and N, ORF1 is recognized to a similar extent by sera from COVID-19 patients and pre–COVID-19 era controls. This suggests that recognition of SARS-CoV-2 ORF1 is a result of cross-reactions from antibodies elicited by exposure to other pathogens, possibly HCoVs. Antibody responses to peptides from membrane glycoprotein (M), ORF3, and ORF9b were occasionally detected in COVID-19 patients.

We also analyzed longitudinal samples from 23 COVID-19 patients. Most patients displayed an antibody response to peptides derived from S or N in the second week after symptom onset, with many displaying an antibody response by the end of the first week (Fig. 2C). The relative strength and onset of the antibody response to S and N differed markedly between individuals, and the initial immune response showed no preference for S or N. The signal intensity of antibodies recognizing SARS-CoV-2 ORF1 epitopes did not increase over time, further suggesting that ORF1 antibodies likely represent a preexisting cross-reactive response.

### Identification of immunogenic regions of SARS-CoV-2 proteins

**Fig. 3:**
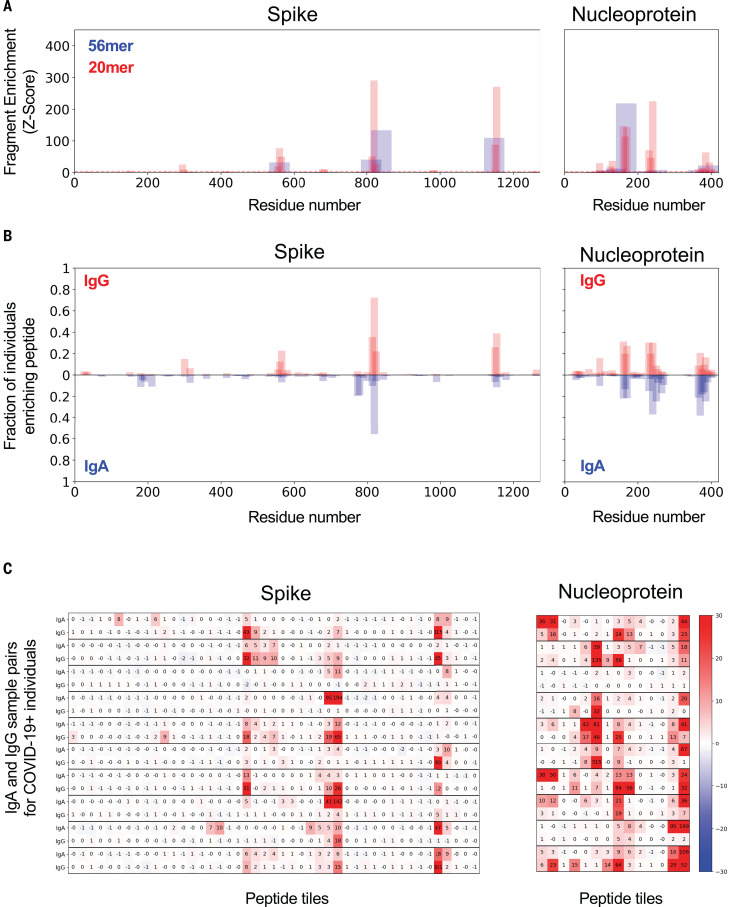
IgG and IgA recognition of immunodominant regions in SARS-CoV-2 spike and nucleoprotein. (**A**) Example response to S and N proteins from a single COVID-19 patient. The *y* axis indicates the strength of enrichment (*z*-score; see Materials and methods) of each 56-mer (blue) or 20-mer (red) peptide recognized by the IgG antibodies present in the serum sample. (**B**) Common responses to S and N proteins across COVID-19 patients. The *y* axis indicates the fraction of COVID-19 patient samples (*n* = 348) enriching each 20-mer peptide with either IgG (top) or IgA (bottom) antibodies. (**C**) Comparison of the IgA and IgG responses in individual COVID-19 patients. Each set of two rows represents the IgG and IgA antibody specificities of a single patient, with data displayed for 10 representative COVID-19 patients. Numeric values indicate the degree of enrichment (*z*-score) of each peptide tiling across the S and N proteins.

To more precisely define the immunogenic regions of the SARS-CoV-2 proteome, we examined the specific 56-mer and 20-mer peptides detected by VirScan in COVID-19 patients compared with those in pre–COVID-19 era controls. An example IgG response from a single patient to SARS-CoV-2 S and N is shown in Fig. 3A. We observed strong concordance between the viral regions enriched by the 56-mer and 20-mer fragments, demonstrating the robustness of VirScan. In many cases, we observed recognition of overlapping 56-mer peptides, indicating an epitope in the common region.

Next, we compared the protein regions recognized by IgG and IgA across COVID-19 patients (Fig. 3B). We identified four regions each in S and N that are recurrently targeted by antibodies from >15% of COVID-19 patients, with additional regions recognized less frequently. Overall, IgG and IgA recognize the same protein regions with similar frequencies across the population. However, when IgG and IgA responses were compared within individuals, we observed considerable divergence (Fig. 3C): Many epitopes were recognized by only IgG, only IgA, or both IgG and IgA within an individual patient. Together, these data suggest that patients generate distinct IgG and IgA antibody responses to SARS-CoV-2, but the targeted regions are largely shared at the population level.

### Machine learning guides the design of a Luminex assay for rapid COVID-19 diagnosis

**Fig. 4:**
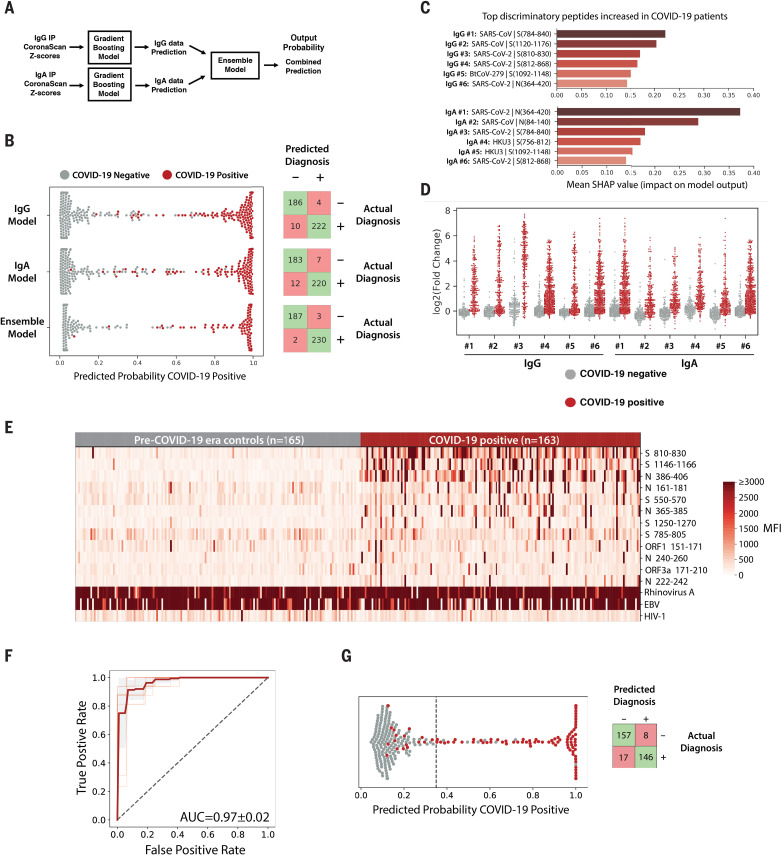
Machine learning models trained on VirScan data discriminate COVID-19–positive and –negative individuals with very high sensitivity and specificity. (**A**) Gradient-boosting machine learning models were trained on IgG and IgA VirScan data from 232 COVID-19 patients and 190 pre–COVID-19 era controls. Separate models were created for the IgG and IgA data, and then a third model (Ensemble) was trained to combine the outputs of the first two. (**B**) The plot shows the predicted probability that each sample is positive for COVID-19. True COVID-19–positive samples are shown as red dots; true COVID-19–negative samples are shown as gray dots. The corresponding confusion matrix for each model is shown on the right. (**C** and **D**) SHAP analysis to identify the most discriminatory peptides informing the models in (B). The chart in (C) summarizes the relative importance of the most discriminatory peptides increased among COVID-19 patients identified by the IgG and IgA gradient-boosting models. The enrichment [log_2_(fold change) of the normalized read counts in the sample IP versus in no-serum control reactions] of each of these peptides across all samples is shown in (D). (**E**) Luminex assay using highly discriminatory SARS-CoV-2 peptides identifies IgG antibody responses in COVID-19 patients but rarely in pre–COVID-19 era controls. Each column represents a COVID-19 patient (*n* = 163) or pre–COVID-19 era control (*n* = 165); each row is a SARS-CoV-2–specific peptide. Peptides containing public epitopes from rhinovirus A, EBV, and HIV-1 served as positive and negative controls. The color scale indicates the median fluorescence intensity (MFI) signals after background subtraction. (**F**) Receiver operating characteristic (ROC) curve for the Luminex assay predicting SARS-CoV-2 infection history, evaluated by 10× cross-validation. The light red lines indicate the ROC curve for each test set, the dark line indicates the average, and the gray region represents ±1 SD. The average area under the curve (AUC) is shown. (**G**) (Left) Predicted probability that each sample is positive for COVID-19, using the Luminex model, as in (B). The dashed line indicates the model threshold. (Right) Confusion matrix for the Luminex model.

To predict SARS-CoV-2 exposure history from VirScan data, we developed a gradient-boosting algorithm (XGBoost) that integrates both IgG and IgA data and predicts current or past COVID-19 with 99.1% sensitivity and 98.4% specificity (Fig. 4, A and B). We used Shapley Additive exPlanations (SHAP)—a method to compute the contribution of each feature of the data to the predictive model (*12*)—to identify peptides from SARS-CoV-2 S and N plus homologous peptides from SARS-CoV and BatCoV-HKU-3 and BatCoV-279 that were highly predictive of SARS-CoV-2 exposure (Fig. 4, C and D).

We leveraged these insights to develop a simple, rapid Luminex-based diagnostic for COVID-19. We chose 12 SARS-CoV-2 peptides predicted by VirScan data and the machine learning model to be highly indicative of SARS-CoV-2 exposure history (table S9). These SARS-CoV-2 peptides, two positive control peptides from rhinovirus A and Epstein-Barr virus (EBV) that are recognized in >80% of seropositive individuals by VirScan (*7*), and a negative control peptide from HIV-1 were coupled to Luminex beads (*13*). We tested 163 COVID-19 patient samples and 165 pre–COVID-19 era controls for IgG reactivity to the Luminex panel. We detected clear responses to SARS-CoV-2 peptides in COVID-19 patient samples but rarely in the pre–COVID-19 era controls (Fig. 4E). Using the Luminex data, we developed a logistic regression model that predicts COVID-19 infection history with 89.6% sensitivity and 95.2% specificity [area under the curve (AUC) = 0.97] (Fig. 4, F and G). A subset of COVID-19–positive samples (*n* = 107) was also examined with an in-house enzyme-linked immunosorbent assay (ELISA) using three SARS-CoV-2 antigens: N, S, and the S receptor-binding domain (RBD). Considering a sample to be positive if it scored above the 99% specificity threshold on any one of the three ELISA antigens, we determined that the sensitivity of the Luminex assay for this subset (88.8%) was similar to that of the ELISA (90.7%) (fig. S1). Among samples run on all three assays, VirScan significantly outperformed both the Luminex and ELISAs (fig. S1, A and C). Notably, our optimal model integrated only three SARS-CoV-2 peptides—residues 386 to 406 of N (N 386-406), residues 810 to 830 of S (S 810-830), and residues 1146 to 1166 of S (S 1146-1166)—which were also the most discriminatory 20-mers in the VirScan data. IgG responses in COVID-19 patients were highly correlated between the Luminex and VirScan assays, providing orthogonal validation of the VirScan data and supporting the prevalence of SARS-CoV-2–induced humoral responses to these regions of S and N (fig. S1D).

### Differential antibody responses to common viruses in hospitalized versus nonhospitalized COVID-19 patients

**Fig. 5:**
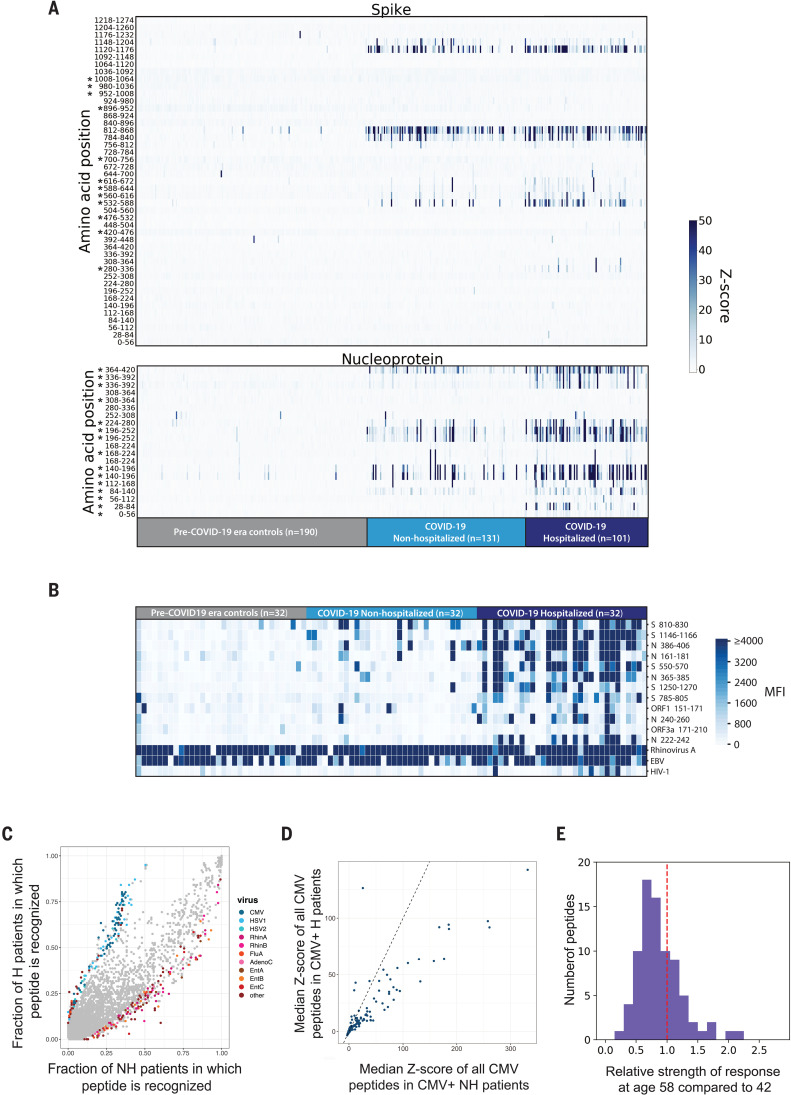
Correlates of COVID-19 severity. (**A**) Differential recognition of peptides from SARS-CoV-2 N and S between COVID-19 nonhospitalized patients (*n* = 131), hospitalized patients (*n* = 101), and pre–COVID-19 era negative controls (*n* = 190). Each column represents a specific patient and each row represents a peptide tile; tiles are labeled by amino acid start and end position and may be duplicated for intervals for which amino acid sequence diversity is represented in the library. Color intensity represents the degree of enrichment (*z*-score) of each peptide in IgG samples. Asterisks indicate peptides that exhibit a significant increase in recognition by sera from hospitalized versus nonhospitalized patients (Kolmogorov-Smirnov test, Bonferroni-corrected *P* value thresholds of 0.001 for S and 0.0025 for N). (**B**) SARS-CoV-2 Luminex assay identifies stronger IgG responses in hospitalized COVID-19 patients than in nonhospitalized COVID-19 patients. Each column represents either a nonhospitalized (*n* = 32) or hospitalized (*n* = 32) COVID-19+ patient or a pre–COVID-19 era control (*n* = 32); each row represents a peptide in the Luminex assay. The color scale indicates the MFI signals after background subtraction. (**C**) All peptides in the VirScan library are plotted by the fraction of nonhospitalized (*x* axis) and hospitalized COVID-19 patient IgG samples (*y* axis) in which they are recognized. A *z*-score threshold of 3.5 was used as an enrichment cutoff to count a peptide as positive. Peptides that exhibit statistically significant associations with hospitalization status are colored by virus of origin (Fisher’s exact test, Bonferroni-corrected *P* value threshold of 8.52 × 10^−7^). All peptides that do not exhibit significant association with hospitalization status are shown in gray. The significant peptides shown are collapsed for high sequence identity. (**D**) All peptides derived from CMV that are present in the VirScan library are plotted by median *z*-score for the nonhospitalized (*x* axis) and hospitalized COVID-19 patients (*y* axis). The line *y* = *x* is shown as a dashed line. (**E**) Reduced recognition of mild disease–associated antigens with age. The histogram shows the relative recognition in healthy donors at age 58 compared with age 42 for each distinct antigen that was more strongly recognized by antibodies in nonhospitalized than hospitalized COVID-19 patients.

We next considered whether differences in the antibody response to SARS-CoV-2 or to other viruses might be associated with the severity of COVID-19. We grouped the COVID-19 patients into two subsets: those who required hospitalization (*n* = 101) and those who did not (*n* = 131). We compared the responses to peptides derived from the SARS-CoV-2 S and N proteins between the hospitalized (H) and nonhospitalized (NH) groups and found that the H group exhibited stronger and broader antibody responses to S and N peptides that might be due to epitope spreading (Fig. 5A). We then analyzed 32 NH COVID-19 samples, 32 H COVID-19 samples, and 32 pre–COVID-19 era negative controls with the Luminex assay and similarly observed that the H group had stronger and broader antibody responses to SARS-CoV-2–specific peptides than did the NH group (Fig. 5B).

VirScan also offers the opportunity to examine the history of previous viral infections and to determine correlates of COVID-19 outcomes. For example, prior viral exposure could provide some protection if cross-reactive neutralizing antibodies or T cell responses are stimulated upon exposure to SARS-CoV-2 (*14*, *15*). Alternatively, cross-reactive antibodies to viral surface proteins could increase the risk of severe disease due to antibody-dependent enhancement (ADE), as has been observed for SARS-CoV (*16*, *17*). Furthermore, exposure to certain viruses could affect the response to SARS-CoV-2 by altering the immune system. To examine these possibilities, we analyzed the virome-wide VirScan data and found that overall, the NH patients exhibited greater responses to individual peptides from common viruses such as rhinoviruses, influenza viruses, and enteroviruses, whereas the H patients displayed more robust responses to peptides from cytomegalovirus (CMV) and herpes simplex virus 1 (HSV-1) (Fig. 5C). These observations may be influenced by demographic differences in the NH and H cohorts, as described below.

We sought to understand whether the differential reactivity to CMV and HSV-1 between the H and NH patients was due to differences in the strength of antibody responses or the prevalence of infection (these viruses are common, but not ubiquitous like rhinoviruses, enteroviruses, and influenza viruses). Using VirScan data, we found that the H group had a higher incidence of both CMV and HSV-1 infection: 82.2% (83 of 101) of the H group were positive for CMV versus 37.4% (49 of 131) of the NH group, whereas 92.1% (93 of 101) of the H group were positive for HSV-1 versus 45.8% (60/131) of the NH group. To examine the relative strength of the antibody responses, we considered only CMV- or HSV-1–seropositive individuals from the NH and H groups: The antibody response to both CMV (Fig. 5D) and HSV-1 (fig. S2) was stronger among the NH individuals. Thus, the differing seroprevalence of CMV and HSV-1 in the NH versus H groups likely explains the results shown in Fig. 5C. We conclude that antibody responses to nearly all viruses, except SARS-CoV-2, were weaker in the H patients than in the NH patients.

These notable differences led us to examine potential demographic covariates between the NH and H groups. We found that age, sex, and race were all significantly associated with COVID-19 severity (fig. S3), as has been reported (*18*, *19*). Older age, male sex, and non-white racial groups were significantly overrepresented in the H group compared with the NH group (fig. S3 and table S3). Furthermore, hospitalized males exhibited stronger responses to N than hospitalized females, whereas nonhospitalized males and females did not exhibit differential responses to any SARS-CoV-2 proteins (fig. S3E). Advanced age is a dominant risk factor for severe COVID-19 and is correlated with reduced immune function (*20*). In light of the age difference between the H (median age: 58) and NH (median age: 42) patients in our cohort, we reasoned that the antigens recognized more strongly in the NH group might reflect more general age-associated changes in humoral immunity. To test this hypothesis, we examined VirScan data for a cohort of 648 healthy, pre-pandemic donors. We characterized the recognition of each NH-associated peptide in subsets of healthy donors representing different age groups and observed a general decline in recognition with age, including a median 19% reduction in recognition from age 42 to 58 (Fig. 5E). These data suggest that age-related changes to the immune system may partially explain the observation of weaker antibody responses to most viruses in the H group. Although it is correlative and potentially influenced by other demographic differences between the NH and H cohorts, the broad age-related diminution in immune system activity that we observed could be an important aspect of the increased severity in the H group.

### Cross reactivity of SARS-CoV-2 epitopes

We returned to the question of epitope cross-reactivity, this time examining antibody responses to the triple-alanine scanning library. For each 56-mer peptide spanning the SARS-CoV-2 proteome, this library contained a collection of scanning mutants: The first mutant peptide encoded three alanines instead of the first three residues, the second mutant peptide contained the three alanines moved one residue downstream, and so on (fig. S4). Antibodies that recognize the wild-type 56-mer peptide will not recognize mutant versions of the peptide containing alanine substitutions at critical residues. Thus, the location of the linear epitope can be deduced by looking for “antibody footprints,” indicated by stretches of alanine mutants missing from the pool of immunoprecipitated phage. The first and last triple-alanine mutations to interfere with binding are expected to start two amino acids before the first residue that is essential for antibody binding and end two amino acids after the last.

**Fig. 6:**
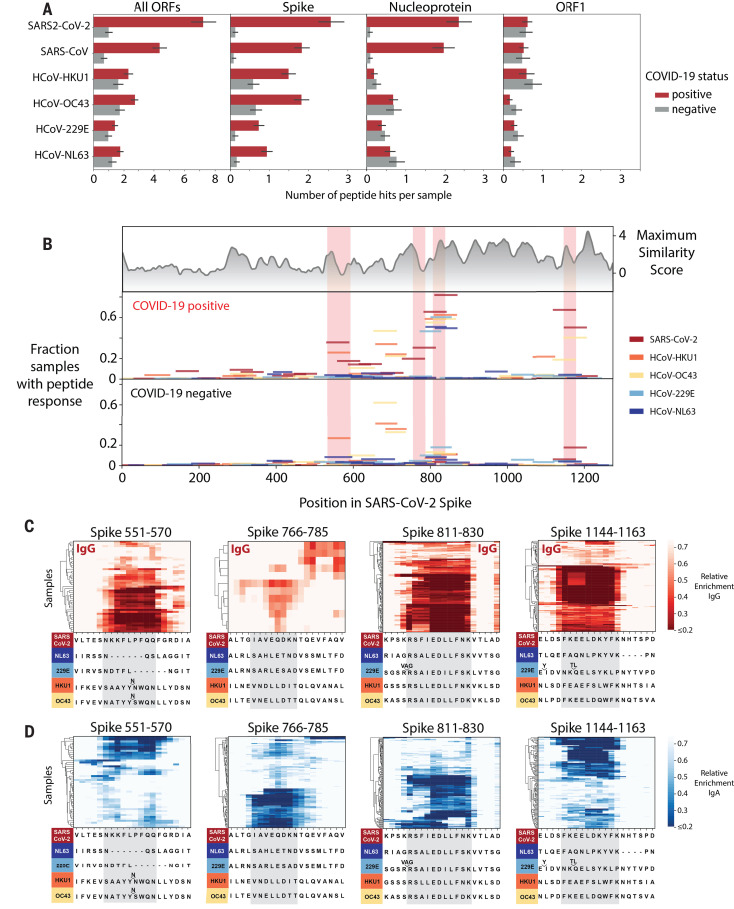
Cross-reactive epitopes among human coronaviruses. (**A**) Bar graphs depict the average number of 56-mer peptides derived from SARS-CoV-2, SARS-CoV, and each of the four common HCoVs that are significantly enriched per sample (IgG IP). Error bars represent 95% confidence intervals. (**B**) Analysis of cross-reactive epitopes for HCoV S proteins. The upper plot shows the similarity of each region of the SARS-CoV-2 S protein to the corresponding region in the four common HCoVs (see Materials and methods). The frequency of peptide recognition is shown in the bottom two plots. Peptides from each virus are indicated by colored lines: The length of each line along the *x* axis indicates the corresponding region of the SARS-CoV-2 S protein covered by each peptide according to a pairwise protein alignment; the height of each line corresponds to the fraction of samples in which that peptide scored in either the IgG or IgA IPs. The epitopes mapped in (C) and (D) are highlighted in pink. (**C** and **D**) Mapping of recurrently recognized SARS-CoV-2 S IgG (C) and IgA (D) epitopes by triple-alanine scanning mutagenesis. Each plot represents a 20–amino acid region of the SARS-CoV-2 S protein within the regions highlighted in (B). Each column of the heatmap corresponds to an amino acid position, and each row represents a sample. The color intensity indicates the average enrichment of 56-mer peptides containing an alanine mutation at that site relative to the median enrichment of all mutants of that 56-mer in each sample. COVID-19 patients with a minimum relative enrichment below 0.6 in the specified window are shown. The amino acid sequence across each region of SARS-CoV-2 S, as well as an alignment of the corresponding sequences in the common HCoVs, is shown below each heatmap. Single-letter abbreviations for the amino acid residues are as follows: A, Ala; C, Cys; D, Asp; E, Glu; F, Phe; G, Gly; H, His; I, Ile; K, Lys; L, Leu; M, Met; N, Asn; P, Pro; Q, Gln; R, Arg; S, Ser; T, Thr; V, Val; W, Trp; and Y, Tyr.

With respect to cross-reactivity, IgG from COVID-19 patients recognized more 56-mer peptides from the common HCoVs HKU1, OC43, 299E, and NL63 than IgG from pre–COVID-19 era controls. This difference is primarily driven by a pronounced increase in recognition of S peptides from the HCoVs and is likely a result of cross-reactivity of antibodies developed during SARS-CoV-2 infection (Fig. 6A).

We mapped the position of all HCoV S peptides that display increased recognition in COVID-19 patient samples onto the SARS-CoV-2 S protein. This revealed four immunodominant regions recognized by >25% of COVID-19 patients (Fig. 6B). Comparing the frequency of peptide recognition between the COVID-19 patients and pre–COVID-19 era controls showed that two of these immunogenic regions in SARS-CoV-2 S are likely to cross-react strongly with homologous regions of other HCoVs, as the frequency of recognition of the HCoV peptides at these regions rises in COVID-19 patients. For instance, peptides from all four seasonal HCoVs that span the region corresponding to residues 811 to 830 of SARS-CoV-2 S are frequently recognized by COVID-19 patients but much less so by pre–COVID-19 era controls, suggesting that this recognition is a result of antibodies developed or boosted in response to SARS-CoV-2 infection. Using triple-alanine scanning mutagenesis (fig. S4), we mapped the antibody footprints in this region to an 11–amino acid stretch that is highly conserved between SARS-CoV-2 and all four common HCoVs, which explains the cross-reactivity (Fig. 6, C and D). Similarly, both SARS-CoV-2 and HCoV-OC43 peptides corresponding to S 1144-1163 are recognized much more frequently by COVID-19 patients than pre–COVID-19 era controls, and triple-alanine-scanning mutagenesis confirmed that the antibody footprints are located within a 10–amino acid stretch conserved between SARS-CoV-2 and HCoV-OC43 but not the other HCoVs. By contrast, the epitope sequences around S 551-570 and S 766-785 are not conserved between SARS-CoV-2 and the seasonal HCoVs, and indeed these epitopes are not cross-reactive. One HCoV-HKU1 peptide spanning S 551-570 scores in both COVID-19 patients and pre–COVID-19 era control samples; however, its frequency of detection is not further boosted in COVID-19 patients, suggesting that the antibody that recognizes the SARS-CoV-2 S 551-570 peptide is distinct from the antibody recognizing the HCoV-HKU1 peptide, consistent with sequence differences at this location (Fig. 6C).

Notably, we detect antibody responses to SARS-CoV-2 S 811-830 in 79.9% of COVID-19 patients. However, we also see responses to the corresponding peptides from OC43 and 229E in ~20% of the pre–COVID-19 era controls, and these responses seem to cross-react with SARS-CoV-2. It is possible that some patients have preexisting antibodies to this region that cross-react and are expanded during SARS-CoV-2 infection. This might explain the very high prevalence of antibody responses to this epitope and suggests that anamnestic responses to seasonal coronaviruses may influence the antibody response to SARS-CoV-2. Of note, this region is located directly after the predicted S2′ cleavage site for SARS-CoV-2 and overlaps the fusion peptide. A recent study showed that adding an excess of the fusion peptide reduced neutralization, implying that an antibody that binds the fusion peptide might contribute to neutralization by interfering with membrane fusion (*21*, *22*). Given the frequency of seroreactivity toward this epitope in COVID-19 patients, it will be important to determine whether the antibodies that recognize this epitope are neutralizing in future studies. If so, the prior presence of antibodies recognizing this epitope may affect the course of COVID-19 and mitigate severity.

### Epitope mapping reveals hundreds of distinct SARS-CoV-2 epitopes, including likely epitopes of neutralizing antibodies

**Fig. 7:**
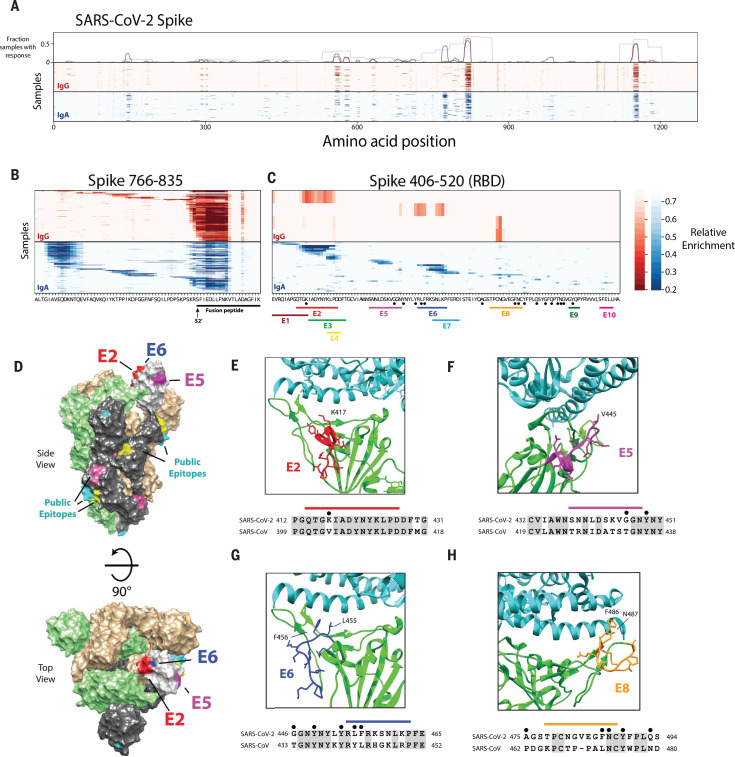
High-resolution mapping of SARS-CoV-2 epitopes. (**A**) Mapping of antibody epitopes in the SARS-CoV-2 S protein using triple-alanine scanning mutagenesis. Each column of the heatmap corresponds to an amino acid position, and each row represents a COVID-19 patient. The color intensity indicates the average enrichment of three triple-alanine mutant 56-mer peptides containing an alanine mutation at that site, relative to the median enrichment of all mutants of that 56-mer. The upper panel shows the fraction of samples that recognized each region of S as mapped by the IgA 56-mer (gray) versus the IgA and IgG triple-alanine scanning data (blue and red, respectively). (**B** and **C**) Detailed plot of the triple-alanine scanning mutagenesis in (A) to show the epitope complexity within two regions: S 766-835 (B) and S 406-520 (C). The amino acid sequence at each position is shown on the *x* axis. In (B), the fusion peptide and predicted S2′ cleavage site are indicated below the sequence (*21*, *22*). In (C) the distinct IgA epitopes identified by the HMM and clustering algorithms are depicted by colored bars. Black dots correspond to ACE2 contact residues in the crystal structure of the RBD receptor complex (6M0J) (*23*). Epitopes in regions E9 and E10 were not picked up by the HMM classifier because of their short length; however, these regions score in multiple samples and correspond to accessible regions in the crystal structure, which suggests that they may represent true epitopes. (**D**) Cryo–electron microscopy (cryo-EM) structure of the partially open SARS-CoV-2 S trimer (6VSB) (*24*), highlighting the locations of the antibody epitopes mapped by triple-alanine scanning mutagenesis. The three S monomers are depicted in tan, green, and gray for the two closed and single open-conformation monomers, respectively. The RBD of the open monomer is show in light gray. Three of the RBD epitopes from (C) that overlap ACE2 contact residues and are resolved in the cryo-EM structure (E2, E5, and E6) are highlighted in red, purple, and blue, respectively. The locations of additional public epitopes that were mapped in at least 10 samples across the IgG and IgA experiments are depicted in yellow, pink, and cyan. (**E** to **H**) The locations of four of the epitope footprints mapped in (C) are shown in relation to the RBD-ACE2 binding interface. The upper image for each panel shows the structure (6M0J) of SARS-CoV-2 RBD (green) in complex with ACE2 (cyan). The E2, E5, E6, and E8 epitopes are highlighted in red, purple, blue, and orange, respectively. Below each structure image is the sequence alignment of the regions of the SARS-CoV-2 and the SARS-CoV S proteins encompassing each epitope. The colored bars indicate each epitope, the black dots indicate residues that directly interact with ACE2 in the crystal structure, and the shaded residues indicate conservation between SARS-CoV-2 and SARS-CoV.

We also used the triple-alanine scanning mutagenesis library to map antibody footprints across the entire SARS-CoV-2 proteome (Fig. 7, fig. S5, and tables S10 to S19). We used a hidden Markov model (HMM) to analyze the mutagenesis data and detect antibody footprints. By integrating signals across stretches of consecutive residues, the HMM successfully distinguished antibody footprints from random noise and thus detected regions containing epitopes with improved sensitivity and far greater resolution than was possible with the 56-mer peptide data alone (see Materials and methods) (figs. S6 and S7 and tables S15 to S18). We performed hierarchical clustering on the antibody footprints identified by the HMM to determine the number of distinct epitopes (here defined as distinct antibody footprints) that we detected across the SARS-CoV-2 proteome (fig. S8 and table S10). Overall, we identified 3103 antibody footprints across 169 COVID-19 patient samples and mapped 823 distinct epitopes (table S19). These epitopes are not evenly distributed along the proteins but rather fall into 303 epitope clusters, each of which contains multiple overlapping epitopes (fig. S8). For example, across the 89 IgA samples that recognized the epitope cluster from S 1135-1165, we identified nine epitopes that overlap but have distinct triple-alanine scanning profiles that indicate distinct antibody-epitope interactions (fig. S8C). Individual epitopes are recognized at a wide range of frequencies in the COVID-19 patients. The average COVID-19 patient sample contained antibodies to ~18 distinct linear epitopes (fig. S9), although this is likely an underestimate of the total epitope count per person, as VirScan does not efficiently detect antibodies recognizing discontinuous (conformational) epitopes (although such antibodies may retain some affinity to linear peptides that constitute the epitope).

The SARS-CoV-2 epitope landscape includes regions recognized by antibodies in a large fraction of COVID-19 patients (“public” epitopes) and regions recognized by antibodies in only one or a few individuals (“private” epitopes). For example, we mapped six distinct epitopes in the region spanning N 151-175 (fig. S5C). One of these epitopes was recognized by nearly one-third of the COVID-19 patients, whereas the rest were detected by <2% of the COVID-19 patients. Similarly, the region spanning S 766-835 contained more than 20 distinct epitopes, including the highly public epitope cluster near S815 and the public epitope cluster near S770 that is preferentially recognized by IgA (Fig. 7B). The public epitope cluster near S770 was recognized in 43% of COVID-19 patient IgA samples but only 4% of COVID-19 patient IgG samples. In another example, we detected at least 20 distinct epitopes within a stretch of just 46 residues in N 363-408, 10 of which were specific to IgA and 2 of which were specific to IgG (fig. S5D). The positions of several public epitope clusters are shown mapped onto the structure of SARS-CoV-2 in fig. S10.

We also mapped at least 12 distinct epitopes in the SARS-CoV-2 RBD, including 5 in the receptor binding motif that binds ACE2, the human receptor for SARS-CoV-2, and 6 that overlap ACE2 binding sites (Fig. 7, C and D, and fig. S6A). For example, S 414-427 (labeled E2 in Fig. 7) spans residue K417 in the RBD; K417 makes a direct contact with the human ACE2 protein in structures of ACE2 bound to the RBD. Thus, antibodies that recognize E2 are likely to block ACE2 binding and have neutralizing activity (Fig. 7E). Epitope S 454-463 (labeled E6 in Fig. 7) also overlaps ACE2 contact residues and partially overlaps the binding site of the neutralizing antibody CB6, which suggests that antibodies recognizing this epitope also have neutralizing potential (*23*–*25*) (Fig. 7G). Several other epitopes also span or are adjacent to critical residues contacted by ACE2 (Fig. 7, F and H). Thus, our data reveal some of the likely binding sites for neutralizing antibodies.

## Discussion

In this study, we used VirScan to analyze sera from COVID-19 patients and pre–COVID-19 era controls to provide an in-depth serological description of antibody responses to SARS-CoV-2. We mapped the landscape of linear epitopes in the SARS-CoV-2 proteome, characterized their specificity or cross-reactivity, and investigated serological and viral exposure history correlates of COVID-19 severity.

### Identification of SARS-CoV-2 epitopes recognized by COVID-19 patients

VirScan detected robust antibody responses to SARS-CoV-2 in COVID-19 patients. These were primarily directed against the S and N proteins, with substantial cross-reactivity to SARS-CoV and milder cross-reactivity with the distantly related MERS-CoV and seasonal HCoVs. Cross-reactive responses to SARS-CoV-2 ORF1 were frequently detected in pre–COVID-19 era controls, suggesting that these result from antibodies induced by other pathogens.

At the population level, most SARS-CoV-2 epitopes were recognized by both IgA and IgG antibodies. We found that individuals often exhibited a “checkerboard” pattern, using either IgG or IgA antibodies against a given epitope. This suggests that a given IgM clone often evolves into either an IgG or IgA antibody, potentially influenced by local signals, and that, within an individual, there may often be a largely monoclonal response to a given epitope.

Examination of the humoral response to SARS-CoV-2 at the epitope level using the triple-alanine scanning mutagenesis library revealed 145 epitopes in S, 116 in N, and 562 across the remainder of the SARS-CoV-2 proteome (table S10). Most S epitopes were located on the surface of the protein or within unstructured regions that often abut, but seldom overlap, glycosylation sites (fig. S11). These epitopes ranged from private to highly public, with one region of S (S 811-830) being recognized by 79.9% of COVID-19 patients. Triple-alanine scanning mutagenesis showed highly conserved antibody footprints for some epitope clusters and diverse antibody footprints for others, indicating varying levels of conservation at the antibody-epitope interface among individuals (fig. S8). Peptides containing public epitopes could be used to isolate and clone antibodies from B cells bearing antigen-specific receptors. If these antibodies are found to lack protective effects or have deleterious effects, these regions could be mutated in future vaccines to divert the immunological response to other regions of S that might have more protective effects. Epitopes also varied in cross-reactivity, which can be explained by the presence or absence of sequence conservation between seasonal HCoVs and SARS-CoV-2 at these regions. Antibodies against several conserved epitopes in HCoVs seemed to be anamnestically boosted in COVID-19 patients. Antibodies recognizing one of these epitopes in the fusion peptide of S2 have been implicated in neutralization, and their presence prior to SARS-CoV-2 infection could mitigate the severity of COVID-19. Collectively, these data help explain why many serological assays for SARS-CoV-2 produce false positives due to preexisting cross-reactive antibodies, some of which may potentially affect the consequences of future SARS-CoV-2 infections.

### Development of SARS-CoV-2 signature peptides for detecting seroconversion by Luminex

Using machine learning models trained on VirScan data, we developed a classifier that predicts SARS-CoV-2 exposure history with 99% sensitivity and 98% specificity. We identified peptides frequently and specifically recognized by COVID-19 patients and used these to create a Luminex assay that predicts SARS-CoV-2 exposure with 90% sensitivity and 95% specificity. Notably, the Luminex assay requires only three peptides to perform comparably to full-antigen ELISAs and could be further optimized in the future. This highlights the utility of VirScan-based serological profiling in the development of rapid and efficient diagnostic assays based on public epitopes.

### Correlates of severity in COVID-19 patients

An important goal is to uncover serological correlates of COVID-19 severity. To this end, we compared cohorts of COVID-19 patients who did (H) or did not (NH) require hospitalization. Using both VirScan and the COVID-19 Luminex assay, we noticed a pronounced and somewhat counterintuitive increase in recognition of peptides derived from the SARS-CoV-2 S and N proteins among the H group, with more extensive epitope spreading. Whether this is a cause or a consequence of severe disease is not clear. Individuals whose innate and adaptive immune responses are not able to quell the infection early may experience a higher viral antigen load, a prolonged period of antibody evolution, and epitope spreading. Consequently, these patients might develop stronger and broader antibody responses to SARS-CoV-2 and could be more likely to have hyperinflammatory reactions such as cytokine storms that increase the probability of hospitalization. We noticed that hospitalized males had more robust antibody responses to SARS-CoV-2 than hospitalized females. This finding may indicate that males in this group are less able to control the virus soon after infection, and it is consistent with reported differences in disease outcomes for males and females (*18*, *19*).

VirScan allowed us to examine viral exposure history, which revealed two notable correlations. First, the seroprevalence of CMV and HSV-1 was much greater in the H group than the NH group. The demographic differences in our relatively small cohort of H versus NH COVID-19 patients make it impossible for us to conclusively determine whether CMV or HSV-1 infection affects disease outcome or is simply associated with other covariates such as age, race, and socioeconomic status. Although CMV prevalence slightly increases with age after 40, it also differs greatly among ethnic and socioeconomic groups (*26*, *27*). CMV is a chronic herpesvirus that is known to have a profound impact on the immune system: It can skew the naïve T-cell repertoire (*28*) and decrease T and B cell function (*29*) and is associated with higher systemic levels of inflammatory mediators (*30*) and increased mortality of people >65 years of age (*31*). The effects of CMV on the immune system could potentially influence COVID-19 outcomes.

The second notable correlation we observed was a substantial decrease in the levels of antibodies that target ubiquitous viruses such as rhinoviruses, enteroviruses, and influenza viruses in COVID-19 H patients compared with NH patients. When we examined only the CMV+ or HSV-1+ individuals in the two groups, we found that the strength of the antibody response to CMV and HSV-1 peptides was also reduced in the H group. We examined the effects of age on viral antibody levels in a pre–COVID-19 era cohort and found a diminution with age in the antibody response against viral peptides differentially recognized between the H and NH groups, consistent with previous studies on the effects of aging on the immune system (*20*). This inferred reduced immunity during aging could affect the severity of COVID-19 outcomes.

In correlative analyses such as these, it is difficult to draw strong conclusions about causality, given the demographic differences in the NH versus H groups. The NH group is younger and has a higher percentage of white and female individuals (average age 42, 66% female) than the H group (average age: 58; 42% female) (fig. S2), consistent with well-documented demographic skews in severely affected COVID-19 patients (*18*, *19*). However, even if age and other demographic factors are covariates, CMV seropositivity and age-related reduction in antibody titers against viral antigens, as described here, could still influence the severity of infection. To test these hypotheses, a much larger cohort of COVID-19 patients with severe and mild disease that could be matched for age, race, and sex is required. Such future studies have the potential to enhance our understanding of the biological mechanisms underlying variable outcomes of COVID-19.

Deep serological profiling can provide a window into the breadth of viral responses, how they differ in patients with diverse outcomes, and how past infections may influence present responses to viral infections. Understanding the epitope landscape of SARS-CoV-2, particularly within S, provides a stepping stone to the isolation and functional dissection of both neutralizing antibodies and antibodies that might exacerbate patient outcomes through ADE and could inform the production of improved diagnostics and vaccines for SARS-CoV-2.

## Materials and methods

### Sources of serum used in this study

#### Cohort 1

Plasma samples were from volunteers recruited at Brigham and Women’s Hospital who had recovered from a confirmed case of COVID-19. All volunteers had a polymerase chain reaction (PCR)–confirmed diagnosis of COVID-19 before being admitted to the study. Volunteers were invited to donate specimens after recovering from their illness and were required to be symptom free for a minimum of 7 days. Participants provided verbal and/or written informed consent and provided blood specimens for analysis. Clinical data, including date of initial symptom onset, symptom type, date of diagnosis, date of symptom cessation, and severity of symptoms, were recorded for all participants, as were results of COVID-19 molecular testing. Participation in these studies was voluntary, and the study protocols have been approved by the respective institutional review boards (IRBs).

#### Cohort 2

Serum samples from patients with PCR-confirmed COVID-19 cases while admitted to the hospital and from patients who were actively enrolled into a prospective study of COVID-19 infection were provided by collaborators from the University of Washington. Residual clinical blood specimens were used. Clinical data, including symptom duration and comorbidities, were extracted from medical records and participant-completed questionnaires. All study procedures have been approved by the University of Washington Institutional Review Board.

#### Cohort 3

Plasma samples were provided by collaborators from Ragon Institute of MGH, MIT and Harvard and Massachusetts General Hospital from study participants in three categories: (i) PCR-confirmed COVID-19 cases while admitted to the hospital; (ii) PCR-confirmed SARS-CoV-2–infected cases seen in an ambulatory setting; and (iii) PCR-confirmed COVID-19 cases in their convalescent stage. All study participants provided verbal and/or written informed consent. Basic data on days since symptom onset were recorded for all participants, as were results of COVID-19 molecular testing. Participation in these studies was voluntary, and the study protocols have been approved by the Partners Institutional Review Board.

#### Cohort 4

Patients were enrolled in the emergency department (ED) at Massachusetts General Hospital in Boston from 15 March to 15 April 2020 during the peak of the COVID-19 surge, with an IRB-approved waiver of informed consent. These included patients 18 years or older with a clinical concern for COVID-19 upon ED arrival and acute respiratory distress with at least one of the following: (i) tachypnea ≥22 breaths per minute, (ii) oxygen saturation ≤92% on room air, (iii) a requirement for supplemental oxygen, or (iv) positive-pressure ventilation. A blood sample was obtained in a 10-ml EDTA tube concurrent with the initial clinical blood draw in the ED. Blood was also drawn on days 3 and 7 if the patient was still hospitalized on those dates. Clinical course was followed to 28 days post-enrollment or until hospital discharge if that occurred after 28 days.

Enrolled individuals who were positive for SARS-CoV-2 were categorized into four outcome groups: (i) requiring mechanical ventilation, with subsequent death; (ii) requiring mechanical ventilation and subsequently recovered; (iii) requiring hospitalization on supplemental oxygen but not mechanical ventilation; and (iv) discharged from ED and not subsequently readmitted with supplemental oxygen. Demographic, past medical, and clinical data were collected and summarized for each outcome group, using medians with interquartile ranges and proportions with 95% confidence intervals, where appropriate.

#### Cohorts 5 and 6

Longitudinal Hopkins cohort: Remnant serum specimens were collected longitudinally from PCR-confirmed COVID-19 patients seen at Johns Hopkins Hospital. Samples were de-identified before analysis, with linked time since onset of symptom information. Specimens were obtained and used in accordance with an approved IRB protocol.

#### Cohorts 7 and 8

Cohorts 7 and 8 were from previous studies (*7*, *8*).

#### Cohort 9

Plasma samples were collected from consenting participants (37 female and 51 male individuals; 18 to 85 years old) of the Partner’s Biobank program at Brigham and Women’s Hospital during the period from July to August 2016. Plasma was harvested after a 10-min 1200xg ficoll density centrifugation from blood that was diluted 1:1 in phosphate buffered saline. Samples were frozen at −30°C in 1-ml aliquots. All samples were collected with Partners Institutional Review Board approval.

### Blood sample collection methods

For cohorts 1 to 3: Blood samples were collected into EDTA (ethylenediamine tetraacetic acid) tubes and spun for 15 min at 2600 rpm according to standard protocol. Plasma was aliquoted into 1.5-ml cryovials and stored at −80°C until analyzed. Only de-identified plasma aliquots including metadata (e.g., days since symptom onset, severity of illness, hospitalization, ICU status, survival) were shared for this study. When appropriate for nonconvalescent samples, plasma or serum was also heat-inactivated at 56°C for 60 min and stored at ≤20°C until analyzed.

For cohort 4: Blood samples were collected in EDTA tubes and processed no more than 3 hours post–blood draw in a biosafety level 2+ laboratory on site. Whole blood was diluted with room temperature RPMI medium in a 1:2 ratio to facilitate cell separation for other analyses using the SepMate PBMC isolation tubes (STEMCELL) containing 16 ml of Ficoll (GE Healthcare). Diluted whole blood was centrifuged at 1200 rcf for 20 min at 20°C. After centrifugation, plasma (5 ml) was pipetted into 15-ml conical tubes and placed on ice during PBMC separation procedures. Plasma was then centrifuged at 1000 rcf for 5 min at 4°C, pipetted in 1.5-ml aliquots into three cryovials (4.5 ml total), and stored at −80°C. For the current study, samples (200 μl) were first randomly allocated onto a 96-well plate on the basis of disease outcome grouping.

### Design and cloning of the SARS-CoV-2 tiling and triple-alanine scanning library

Multiple VirScan libraries were constructed as described below. We created ~200-nt oligos encoding peptide sequences 56 amino acids in length, tiled with 28–amino acid overlap through the proteomes of all coronaviruses known to infect humans, including HCoV-NL63, HCoV-229E, HCoV-OC43, HCoV- HKU1, SARS-CoV, MERS-CoV, and SARS-CoV-2, as well as three closely related bat viruses (BatCoV-Rp3, BatCoV-HKU3, and BatCoV-279). For SARS-CoV-2, we included a number of coding variants available in early sequencing of the viruses. For SARS-CoV-2, we additionally made a 20–amino acid peptide library tiling every five amino acids. Additionally, for SARS-CoV-2 we made triple-alanine mutant sequences scanning through all 56-mer peptides. Non-alanine amino acids were mutated to alanine, and alanines were mutated to glycine. Each peptide in all three libraries was encoded in two distinct ways such that there were duplicate peptides that could be distinguished by DNA sequencing. We reverse-translated the peptide sequences into DNA sequences that were codon-optimized for expression in *Escherichia coli*, that lacked restriction sites used in downstream cloning steps (EcoRI and XhoI), and that were distinct in the 50 nt at the 5′ end to allow for unambiguous mapping of the sequencing results. Then we added adapter sequences to the 5′ and 3′ ends to form the final oligonucleotide sequences (table S1): These adapter sequences facilitated downstream PCR and cloning steps. Different adapters were added to each sublibrary so that they could be amplified separately. The resulting sequences were synthesized on a releasable DNA microarray (Agilent). We PCR-amplified the DNA oligo library with the primers shown below, digested the product with EcoRI and XhoI, and cloned it into the EcoRI/SalI site of the T7FNS2 vector (*5*). We packaged the resultant library into T7 bacteriophage using the T7 Select Packaging Kit (EMD Millipore) and amplified the library according to the manufacturer’s protocol.

Primers used for analysis of the different libraries employed:

CoV 56-mer library:

5′ adapter: 5′-GAATTCGGAGCGGT-3′

3′ adapter: 5′-CACTGCACTCGAGA-3′

Forward primer: 5′-AATGATACGGCGTGAATTCGGAGCGGT-3′

Reverse primer: 5′-CAAGCAGAAGACGTCTCGAGTGCAGTG-3′

SARS CoV-2 triple-alanine scanning library:

5′ adapter: 5′-GAATTCCGCTGCGT-3′

3′ adapter: 5′-CAGGGAAGAGCTCG-3′

Forward primer: 5′-AATGATACGGCGGGAATTCCGCTGCGT-3′

Reverse primer: 5′-CAAGCAGAAGACTCGAGCTCTTCCCTG-3′

SARS-CoV-2 20-mer library:

5′ adapter: 5′-GAATTCCGCTGCGT-3′

3′ adapter: 5′-GTACTATACCTACGGAAGGCTCG-3′

Forward primer: 5′-AATGATACGGCGGGAATTCCGCTGCGT-3′

Reverse primer: 5′-TATCTCGCATAGCGCATATACTCGAGCCTTCCGTAGGTATAGTAC-3′

### Phage immunoprecipitation and sequencing

We performed phage IP and sequencing as described previously or with slight modifications (*5*–*8*). For the IgA and IgG chain isotype-specific IPs, we substituted magnetic protein A and protein G Dynabeads (Invitrogen) with 6 μg of Mouse Anti-Human IgG Fc-BIOT (Southern Biotech) or 4 μg of Goat Anti-Human IgA-BIOT (Southern Biotech) antibodies. We added these antibodies to the phage and serum mixture and incubated the reactions overnight a 4°C. Next, we added 25 or 20 μl of Pierce Streptavidin Magnetic Beads (Thermo-Fisher) to the IgG or IgA reactions, respectively, and incubated the reactions for 4 hours at room temperature, then continued with the washing steps and the remainder of the protocol, as previously described (*5*–*8*).

### Machine learning classifiers

Gradient-boosting classifier models for the VirScan data were generated using the XGBoost algorithm (version 1.0.2). Classifier models were trained to discriminate either COVID-19+ and COVID-19− patients (*n* = 232 and 190, respectively) or severe disease and mild disease (*n* = 101 hospitalized patients and *n* = 131 nonhospitalized patients). Two models were generated in each case, one using the *z*-scores for each VirScan peptide from the IgG IP as input features, and the other using the *z*-scores for each VirScan peptide from the IgA IP as input features. Additionally, a third logistic regression classifier was trained on the output probabilities from the IgG and IgA models to generate a combined prediction. The performance of each of the three model was assessed using a 20-fold cross-validation procedure, whereby predictions for each 5% of the data points were generated from a model trained on the remaining 95%. The SHAP package was used to identify the top discriminatory peptide features from each of the XGBoost models. The logistic regression models for the Luminex data were generated using the scikit-learn python package. The raw median fluorescence intensity (MFI) values were preprocessed using the RobustScalar function, then a logistic regression model was trained using the three most discriminate SARS-CoV-2 peptides. The model performance was quantified by 10-fold cross-validation.

### High-resolution epitope identification and clustering

For each position in the 56-mer, the relative enrichment for each amino acid was calculated as the mean fold change of the three mutant peptides containing an alanine mutation at that location relative to the median fold change of all alanine mutants for the 56-mer. Overlapping 56-mers were combined by taking the minimum value at each shared position to account for the possibility that an epitope is interrupted in one of the tiles by the peptide junction. To map the boundaries of antibody footprints from the triple-alanine scanning data for each sample we used the hmmlearn python package to develop a three-state HMM assuming a Gaussian distribution of relative enrichment emissions for each state. Mapped antibody footprints smaller than five amino acids in length were removed from the subsequent analysis. Next, we performed a two-step hierarchical clustering procedure to identify the number of distinct epitopes. First, for each protein all antibody footprints identified across the 169 COVID-19+ patient samples were clustered based on the start and stop locations predicted by the HMM classifier to generate epitope clusters. Next, to identify distinct epitopes, we performed an additional step of hierarchical clustering on the samples with epitopes within each epitope cluster based on the relative enrichment values of the triple-alanine mutants spanning the epitope (fig. S8).

### Similarity-score calculation

Pairwise alignments were generated for the S proteins of SARS-CoV-2 and each of the four common HCoVs. Similarity scores were calculated separately for a 21–amino acid window centered at each position of the SARS-CoV-2 S protein. The mean similarity score between SARS-CoV-2 and the corresponding sequence of the other HCoV was calculated for each window using the BLOSUM62 substitution matrix with a gap opening and extending penalty of −10 and −1, respectively. The maximum similarity was score was calculated as the maximum value among the pairwise similarity scores between SARS-CoV-2 and each of the four common HCoVs for the sliding window centered at each position.

### Luminex multiplex peptide epitope serology assays

Multiplexed SARS-CoV-2 peptide epitope assays were built using the peptides listed in table S9. Peptides were synthesized by the Ragon/MGH Peptide Core Facility with a Proparglyglycine (Pra, X) moiety in the N terminus to facilitate cross-linking to Luminex beads using a “click” chemistry strategy as described previously (*13*). In brief, Luminex beads were first functionalized with amine-PEG4-azide and then reacted with the peptides to generate 20 different Luminex beads with attached peptides. Luminex bead–based serology assays were performed in 96-well U-bottom polypropylene plates using PBS + 0.1% bovine serum albumin as the assay buffer. Bead washes were done using PBS + 0.05% Triton X-100 by incubation for 1 min on a strong magnetic plate (Millipore-Sigma, Burlington, MA). All assay incubation times were 20 min. In the first step, beads were incubated with 20 μl of plasma samples. Samples used for the classifier were diluted 1:100, samples used to compare disease severity were diluted 1:300. After a wash step, bound IgA or IgG was detected by adding 40 μl of biotin-labeled anti-IgA or IgG antibodies at 0.1 μg/ml (Southern Biotechnology, Birmingham, AL). Next, 40 μl of phycoerythrin (PE)–labeled streptavidin (0.2 μg/ml) (Biolegend, San Diego, CA) and assay plates were analyzed on a Luminex FLEXMAP 3D instrument (Luminex Corporation, Austin, Texas) to generate MFI values to quantify peptide-specific IgA or IgG levels.

### ELISA serology assays

ELISAs were performed separately using the SARS-CoV-2 N protein, S protein, or the S receptor-binding domain (RBD). 96-well plates were coated with antigen overnight. The plates were then blocked in PBS + 3% BSA. After washing with PBS + 0.05% Tween-20, the plasma sample were diluted 1:100, added to the plates and incubated overnight at 4°C. After incubation, the plates were washed three times with PBS + 0.05% Tween-20. The bound IgG was detected by adding anti-human IgG-alkaline phosphatase (Southern Biotech, Birmingham, AL) and incubating for 90 min at room temperature. The plates were washed an additional three times, after which p-nitrophenyl phosphate solution (1.6 mg/ml in 0.1 M glycine, 1 mM ZnCl_2_, 1 mM MgCl_2_, pH 10.4) was added to each well and allowed to develop for 2 hours. Bound IgG was quantified by measuring the OD405, and the reported values were calculated as the fold change over the pre–COVID-19 controls.

## MGH COVID-19 Collection & Processing Team participants

**Collection Team:** Kendall Lavin-Parsons^1^, Blair Parry^1^, Brendan Lilley^1^, Carl Lodenstein^1^, Brenna McKaig^1^, Nicole Charland^1^, Hargun Khanna^1^, Justin Margolin^1^

**Processing Team:** Anna Gonye^2^, Irena Gushterova^2^, Tom Lasalle^2^, Nihaarika Sharma^2^, Brian C. Russo^3^, Maricarmen Rojas-Lopez^3^, Moshe Sade-Feldman^4^, Kasidet Manakongtreecheep^4^, Jessica Tantivit^4^, Molly Fisher Thomas^4^

**Massachusetts Consortium on Pathogen Readiness:**Betelihem A. Abayneh^5^, Patrick Allen^5^, Diane Antille^5^, Katrina Armstrong^5^, Siobhan Boyce^5^, Joan Braley^5^, Karen Branch^5^, Katherine Broderick^5^, Julia Carney^5^, Andrew Chan^5^, Susan Davidson^5^, Michael Dougan^5^, David Drew^5^, Ashley Elliman^5^, Keith Flaherty^5^, Jeanne Flannery^5^, Pamela Forde^5^, Elise Gettings^5^, Amanda Griffin^5^, Sheila Grimmel^5^, Kathleen Grinke^5^, Kathryn Hall^5^, Meg Healy^5^, Deborah Henault^5^, Grace Holland^5^, Chantal Kayitesi^5^, Vlasta LaValle^5^, Yuting Lu^5^, Sarah Luthern^5^, Jordan Marchewka (Schneider)^5^, Brittani Martino^5^, Roseann McNamara^5^, Christian Nambu^5^, Susan Nelson^5^, Marjorie Noone^5^, Christine Ommerborn^5^, Lois Chris Pacheco^5^, Nicole Phan^5^, Falisha A. Porto^5^, Edward Ryan^5^, Kathleen Selleck^5^, Sue Slaughenhaupt^5^, Kimberly Smith Sheppard^5^, Elizabeth Suschana^5^, Vivine Wilson^5^, Galit Alter^6^, Alejandro Balazs^6^, Julia Bals^6^, Max Barbash^6^, Yannic Bartsch^6^, Julie Boucau^6^, Josh Chevalier^6^, Fatema Chowdhury^6^, Kevin Einkauf^6^, Jon Fallon^6^, Liz Fedirko^6^, Kelsey Finn^6^, Pilar Garcia-Broncano^6^, Ciputra Hartana^6^, Chenyang Jiang^6^, Paulina Kaplonek^6^, Marshall Karpell^6^, Evan C. Lam^6^, Kristina Lefteri^6^, Xiaodong Lian^6^, Mathias Lichterfeld^6^, Daniel Lingwood^6^, Hang Liu^6^, Jinqing Liu^6^, Natasha Ly^6^, Ashlin Michell^6^, Ilan Millstrom^6^, Noah Miranda^6^, Claire O’Callaghan^6^, Matthew Osborn^6^, Shiv Pillai^6^, Yelizaveta Rassadkina^6^, Alexandra Reissis^6^, Francis Ruzicka^6^, Kyra Seiger^6^, Libera Sessa^6^, Christianne Sharr^6^, Sally Shin^6^, Nishant Singh^6^, Weiwei Sun^6^, Xiaoming Sun^6^, Hannah Ticheli^6^, Alicja Trocha-Piechocka^6^, Daniel Worrall^6^, Alex Zhu^6^, George Daley^7^, David Golan^7^, Howard Heller^7^, Arlene Sharpe^7^, Nikolaus Jilg^8^, Alex Rosenthal^8^, Colline Wong^8^

^1^Department of Emergency Medicine, Massachusetts General Hospital, Boston, MA, USA. ^2^Massachusetts General Hospital Cancer Center, Boston, MA, USA. ^3^Division of Infectious Diseases, Department of Medicine, Massachusetts General Hospital, Boston, MA, USA. ^4^Massachusetts General Hospital Center for Immunology and Inflammatory Diseases, Boston, MA, USA. ^5^Massachusetts General Hospital, Boston, MA, USA. ^6^Ragon Institute of MGH, MIT and Harvard, Cambridge, MA, USA. ^7^Harvard Medical School, Boston, MA, USA. ^8^Brigham and Women’s Hospital, Boston, MA, USA.

## Supplementary Materials

science.sciencemag.org/content/370/6520/eabd4250/suppl/DC1 Figs. S1 to S11 Tables S1 to S19 References (*37*–*39*) MDAR Reproducibility Checklist

## Appendix

View/request a protocol for this paper from *Bio-protocol*.

## References

[1] J. Cui, F. Li, Z.-L. Shi, Origin and evolution of pathogenic coronaviruses. Nat. Rev. Microbiol. 17, 181–192 (2019). 10.1038/s41579-018-0118-9 30531947 PMC7097006

[2] D.-G. Ahn, H.-J. Shin, M.-H. Kim, S. Lee, H.-S. Kim, J. Myoung, B.-T. Kim, S.-J. Kim, Current Status of Epidemiology, Diagnosis, Therapeutics, and Vaccines for Novel Coronavirus Disease 2019 (COVID-19). J. Microbiol. Biotechnol. 30, 313–324 (2020). 10.4014/jmb.2003.03011 32238757 PMC9728410

[3] COVID-19 Dashboard by the Center for Systems Science and Engineering (CSSE) at Johns Hopkins University (JHU); https://coronavirus.jhu.edu/map.html. 10.1016/S1473-3099(22)00434-0 PMC9432867 36057267

[4] K. Yuki, M. Fujiogi, S. Koutsogiannaki, COVID-19 pathophysiology: A review. Clin. Immunol. 215, 108427 (2020). 10.1016/j.clim.2020.108427 32325252 PMC7169933

[5] H. B. Larman, Z. Zhao, U. Laserson, M. Z. Li, A. Ciccia, M. A. M. Gakidis, G. M. Church, S. Kesari, E. M. Leproust, N. L. Solimini, S. J. Elledge, Autoantigen discovery with a synthetic human peptidome. Nat. Biotechnol. 29, 535–541 (2011). 10.1038/nbt.1856 21602805 PMC4169279

[6] D. Mohan, D. L. Wansley, B. M. Sie, M. S. Noon, A. N. Baer, U. Laserson, H. B. Larman, PhIP-Seq characterization of serum antibodies using oligonucleotide-encoded peptidomes. Nat. Protoc. 13, 1958–1978 (2018). 10.1038/s41596-018-0025-6 30190553 PMC6568263

[7] G. J. Xu, T. Kula, Q. Xu, M. Z. Li, S. D. Vernon, T. Ndung’u, K. Ruxrungtham, J. Sanchez, C. Brander, R. T. Chung, K. C. O’Connor, B. Walker, H. B. Larman, S. J. Elledge, Comprehensive serological profiling of human populations using a synthetic human virome. Science 348, aaa0698 (2015). 10.1126/science.aaa0698 26045439 PMC4844011

[8] M. J. Mina, T. Kula, Y. Leng, M. Li, R. D. de Vries, M. Knip, H. Siljander, M. Rewers, D. F. Choy, M. S. Wilson, H. B. Larman, A. N. Nelson, D. E. Griffin, R. L. de Swart, S. J. Elledge, Measles virus infection diminishes preexisting antibodies that offer protection from other pathogens. Science 366, 599–606 (2019). 10.1126/science.aay6485 31672891 PMC8590458

[9] National Center for Biotechnology Information, National Library of Medicine, Protein database (2004) [cited 29 February 2020]; www.ncbi.nlm.nih.gov/protein/.

[10] P. Zhou, X.-L. Yang, X.-G. Wang, B. Hu, L. Zhang, W. Zhang, H.-R. Si, Y. Zhu, B. Li, C.-L. Huang, H.-D. Chen, J. Chen, Y. Luo, H. Guo, R.-D. Jiang, M.-Q. Liu, Y. Chen, X.-R. Shen, X. Wang, X.-S. Zheng, K. Zhao, Q.-J. Chen, F. Deng, L.-L. Liu, B. Yan, F.-X. Zhan, Y.-Y. Wang, G.-F. Xiao, Z.-L. Shi, A pneumonia outbreak associated with a new coronavirus of probable bat origin. Nature 579, 270–273 (2020). 10.1038/s41586-020-2012-7 32015507 PMC7095418

[11] G. J. Gorse, G. B. Patel, J. N. Vitale, T. Z. O’Connor, Prevalence of antibodies to four human coronaviruses is lower in nasal secretions than in serum. Clin. Vaccine Immunol. 17, 1875–1880 (2010). 10.1128/CVI.00278-10 20943876 PMC3008199

[12] S. M. Lundberg, G. Erion, H. Chen, A. DeGrave, J. M. Prutkin, B. Nair, R. Katz, J. Himmelfarb, N. Bansal, S.-I. Lee, From local explanations to global understanding with explainable AI for trees. Nat. Mach. Intell. 2, 56–67 (2020). 10.1038/s42256-019-0138-9 32607472 PMC7326367

[13] M. B. Coppock, D. N. Stratis-Cullum, A universal method for the functionalization of dyed magnetic microspheres with peptides. Methods 158, 12–16 (2019). 10.1016/j.ymeth.2019.01.014 30707950

[14] A. Grifoni, D. Weiskopf, S. I. Ramirez, J. Mateus, J. M. Dan, C. R. Moderbacher, S. A. Rawlings, A. Sutherland, L. Premkumar, R. S. Jadi, D. Marrama, A. M. de Silva, A. Frazier, A. F. Carlin, J. A. Greenbaum, B. Peters, F. Krammer, D. M. Smith, S. Crotty, A. Sette, Targets of T Cell Responses to SARS-CoV-2 Coronavirus in Humans with COVID-19 Disease and Unexposed Individuals. Cell 181, 1489–1501.e15 (2020). 10.1016/j.cell.2020.05.015 32473127 PMC7237901

[15] N. Le Bert, A. T. Tan, K. Kunasegaran, C. Y. L. Tham, M. Hafezi, A. Chia, M. H. Y. Chng, M. Lin, N. Tan, M. Linster, W. N. Chia, M. I.-C. Chen, L.-F. Wang, E. E. Ooi, S. Kalimuddin, P. A. Tambyah, J. G.-H. Low, Y.-J. Tan, A. Bertoletti, SARS-CoV-2-specific T cell immunity in cases of COVID-19 and SARS, and uninfected controls. Nature 584, 457–462 (2020). 10.1038/s41586-020-2550-z 32668444

[16] Y. Wan, J. Shang, S. Sun, W. Tai, J. Chen, Q. Geng, L. He, Y. Chen, J. Wu, Z. Shi, Y. Zhou, L. Du, F. Li, Molecular Mechanism for Antibody-Dependent Enhancement of Coronavirus Entry. J. Virol. 94, e02015-19 (2020). 10.1128/JVI.02015-19 31826992 PMC7022351

[17] S.-F. Wang, S.-P. Tseng, C.-H. Yen, J.-Y. Yang, C.-H. Tsao, C.-W. Shen, K.-H. Chen, F.-T. Liu, W.-T. Liu, Y.-M. A. Chen, J. C. Huang, Antibody-dependent SARS coronavirus infection is mediated by antibodies against spike proteins. Biochem. Biophys. Res. Commun. 451, 208–214 (2014). 10.1016/j.bbrc.2014.07.090 25073113 PMC7092860

[18] M. Webb Hooper, A. M. Nápoles, E. J. Pérez-Stable, COVID-19 and Racial/Ethnic Disparities. JAMA 323, 2466–2467 (2020). 10.1001/jama.2020.8598 32391864 PMC9310097

[19] S. Garg, L. Kim, M. Whitaker, A. O’Halloran, C. Cummings, R. Holstein, M. Prill, S. J. Chai, P. D. Kirley, N. B. Alden, B. Kawasaki, K. Yousey-Hindes, L. Niccolai, E. J. Anderson, K. P. Openo, A. Weigel, M. L. Monroe, P. Ryan, J. Henderson, S. Kim, K. Como-Sabetti, R. Lynfield, D. Sosin, S. Torres, A. Muse, N. M. Bennett, L. Billing, M. Sutton, N. West, W. Schaffner, H. K. Talbot, C. Aquino, A. George, A. Budd, L. Brammer, G. Langley, A. J. Hall, A. Fry, Hospitalization Rates and Characteristics of Patients Hospitalized with Laboratory-Confirmed Coronavirus Disease 2019 - COVID-NET, 14 States, March 1-30, 2020. Morb. Mortal. Wkly. Rep. 69, 458–464 (2020). 10.15585/mmwr.mm6915e3 32298251 PMC7755063

[20] E. Montecino-Rodriguez, B. Berent-Maoz, K. Dorshkind, Causes, consequences, and reversal of immune system aging. J. Clin. Invest. 123, 958–965 (2013). 10.1172/JCI64096 23454758 PMC3582124

[21] C. M. Poh, G. Carissimo, B. Wang, S. N. Amrun, C. Y.-P. Lee, R. S.-L. Chee, S.-W. Fong, N. K.-W. Yeo, W.-H. Lee, A. Torres-Ruesta, Y.-S. Leo, M. I.-C. Chen, S.-Y. Tan, L. Y. A. Chai, S. Kalimuddin, S. S. G. Kheng, S.-Y. Thien, B. E. Young, D. C. Lye, B. J. Hanson, C.-I. Wang, L. Renia, L. F. P. Ng, Two linear epitopes on the SARS-CoV-2 spike protein that elicit neutralising antibodies in COVID-19 patients. Nat. Commun. 11, 2806 (2020). 10.1038/s41467-020-16638-2 32483236 PMC7264175

[22] A. C. Walls, Y.-J. Park, M. A. Tortorici, A. Wall, A. T. McGuire, D. Veesler, Structure, Function, and Antigenicity of the SARS-CoV-2 Spike Glycoprotein. Cell 181, 281–292.e6 (2020). 10.1016/j.cell.2020.02.058 32155444 PMC7102599

[23] J. Lan, J. Ge, J. Yu, S. Shan, H. Zhou, S. Fan, Q. Zhang, X. Shi, Q. Wang, L. Zhang, X. Wang, Structure of the SARS-CoV-2 spike receptor-binding domain bound to the ACE2 receptor. Nature 581, 215–220 (2020). 10.1038/s41586-020-2180-5 32225176

[24] D. Wrapp, N. Wang, K. S. Corbett, J. A. Goldsmith, C.-L. Hsieh, O. Abiona, B. S. Graham, J. S. McLellan, Cryo-EM structure of the 2019-nCoV spike in the prefusion conformation. Science 367, 1260–1263 (2020). 10.1126/science.abb2507 32075877 PMC7164637

[25] R. Shi, C. Shan, X. Duan, Z. Chen, P. Liu, J. Song, T. Song, X. Bi, C. Han, L. Wu, G. Gao, X. Hu, Y. Zhang, Z. Tong, W. Huang, W. J. Liu, G. Wu, B. Zhang, L. Wang, J. Qi, H. Feng, F.-S. Wang, Q. Wang, G. F. Gao, Z. Yuan, J. Yan, A human neutralizing antibody targets the receptor-binding site of SARS-CoV-2. Nature 584, 120–124 (2020). 10.1038/s41586-020-2381-y 32454512

[26] R. Lachmann, A. Loenenbach, T. Waterboer, N. Brenner, M. Pawlita, A. Michel, M. Thamm, C. Poethko-Müller, O. Wichmann, M. Wiese-Posselt, Cytomegalovirus (CMV) seroprevalence in the adult population of Germany. PLOS ONE 13, e0200267 (2018). 10.1371/journal.pone.0200267 30044826 PMC6059406

[27] S. L. Bate, S. C. Dollard, M. J. Cannon, Cytomegalovirus seroprevalence in the United States: The national health and nutrition examination surveys, 1988-2004. Clin. Infect. Dis. 50, 1439–1447 (2010). 10.1086/652438 20426575 PMC11000537

[28] P. Klenerman, P. R. Dunbar, CMV and the art of memory maintenance. Immunity 29, 520–522 (2008). 10.1016/j.immuni.2008.09.008 18957264

[29] G. Pawelec, S. Koch, H. Griesemann, A. Rehbein, K. Hähnel, C. Gouttefangeas, Immunosenescence, suppression and tumour progression. Cancer Immunol. Immunother. 55, 981–986 (2006). 10.1007/s00262-005-0109-3 16333622 PMC11030987

[30] J. L. Craigen, K. L. Yong, N. J. Jordan, L. P. MacCormac, J. Westwick, A. N. Akbar, J. E. Grundy, Human cytomegalovirus infection up-regulates interleukin-8 gene expression and stimulates neutrophil transendothelial migration. Immunology 92, 138–145 (1997). 10.1046/j.1365-2567.1997.00310.x 9370936 PMC1363993

[31] G. M. Savva, A. Pachnio, B. Kaul, K. Morgan, F. A. Huppert, C. Brayne, P. A. H. Moss; Medical Research Council Cognitive Function and Ageing Study, Cytomegalovirus infection is associated with increased mortality in the older population. Aging Cell 12, 381–387 (2013). 10.1111/acel.12059 23442093

[32] J. F. W. Chan, S. K. P. Lau, K. K. W. To, V. C. C. Cheng, P. C. Y. Woo, K.-Y. Yuen, Middle East respiratory syndrome coronavirus: Another zoonotic betacoronavirus causing SARS-like disease. Clin. Microbiol. Rev. 28, 465–522 (2015). 10.1128/CMR.00102-14 25810418 PMC4402954

[33] N. Saitou, M. Nei, The neighbor-joining method: A new method for reconstructing phylogenetic trees. Mol. Biol. Evol. 4, 406–425 (1987). 3447015 10.1093/oxfordjournals.molbev.a040454

[34] S. Kumar, G. Stecher, M. Li, C. Knyaz, K. Tamura, MEGA X: Molecular Evolutionary Genetics Analysis across Computing Platforms. Mol. Biol. Evol. 35, 1547–1549 (2018). 10.1093/molbev/msy096 29722887 PMC5967553

[35] K. Tamura, M. Nei, S. Kumar, Prospects for inferring very large phylogenies by using the neighbor-joining method. Proc. Natl. Acad. Sci. U.S.A. 101, 11030–11035 (2004). 10.1073/pnas.0404206101 15258291 PMC491989

[36] D. E. Gordon, G. M. Jang, M. Bouhaddou, J. Xu, K. Obernier, K. M. White, M. J. O’Meara, V. V. Rezelj, J. Z. Guo, D. L. Swaney, T. A. Tummino, R. Hüttenhain, R. M. Kaake, A. L. Richards, B. Tutuncuoglu, H. Foussard, J. Batra, K. Haas, M. Modak, M. Kim, P. Haas, B. J. Polacco, H. Braberg, J. M. Fabius, M. Eckhardt, M. Soucheray, M. J. Bennett, M. Cakir, M. J. McGregor, Q. Li, B. Meyer, F. Roesch, T. Vallet, A. Mac Kain, L. Miorin, E. Moreno, Z. Z. C. Naing, Y. Zhou, S. Peng, Y. Shi, Z. Zhang, W. Shen, I. T. Kirby, J. E. Melnyk, J. S. Chorba, K. Lou, S. A. Dai, I. Barrio-Hernandez, D. Memon, C. Hernandez-Armenta, J. Lyu, C. J. P. Mathy, T. Perica, K. B. Pilla, S. J. Ganesan, D. J. Saltzberg, R. Rakesh, X. Liu, S. B. Rosenthal, L. Calviello, S. Venkataramanan, J. Liboy-Lugo, Y. Lin, X.-P. Huang, Y. Liu, S. A. Wankowicz, M. Bohn, M. Safari, F. S. Ugur, C. Koh, N. S. Savar, Q. D. Tran, D. Shengjuler, S. J. Fletcher, M. C. O’Neal, Y. Cai, J. C. J. Chang, D. J. Broadhurst, S. Klippsten, P. P. Sharp, N. A. Wenzell, D. Kuzuoglu-Ozturk, H.-Y. Wang, R. Trenker, J. M. Young, D. A. Cavero, J. Hiatt, T. L. Roth, U. Rathore, A. Subramanian, J. Noack, M. Hubert, R. M. Stroud, A. D. Frankel, O. S. Rosenberg, K. A. Verba, D. A. Agard, M. Ott, M. Emerman, N. Jura, M. von Zastrow, E. Verdin, A. Ashworth, O. Schwartz, C. d’Enfert, S. Mukherjee, M. Jacobson, H. S. Malik, D. G. Fujimori, T. Ideker, C. S. Craik, S. N. Floor, J. S. Fraser, J. D. Gross, A. Sali, B. L. Roth, D. Ruggero, J. Taunton, T. Kortemme, P. Beltrao, M. Vignuzzi, A. García-Sastre, K. M. Shokat, B. K. Shoichet, N. J. Krogan, A SARS-CoV-2 protein interaction map reveals targets for drug repurposing. Nature 583, 459–468 (2020). 10.1038/s41586-020-2286-9 32353859 PMC7431030

[37] P. Zhao, J. L. Praissman, O. C. Grant, Y. Cai, T. Xiao, K. E. Rosenbalm, K. Aoki, B. P. Kellman, R. Bridger, D. H. Barouch, M. A. Brindley, N. E. Lewis, M. Tiemeyer, B. Chen, R. J. Woods, L. Wells, Virus-Receptor Interactions of Glycosylated SARS-CoV-2 Spike and Human ACE2 Receptor. Cell Host Microbe 28, 1–16 (2020). 32841605 10.1016/j.chom.2020.08.004 PMC7443692

[38] M. C. Jespersen, B. Peters, M. Nielsen, P. Marcatili, BepiPred-2.0: Improving sequence-based B-cell epitope prediction using comformational epitopes. Nucleic Acids Res. 45, W24–W29 (2017). 10.1093/nar/gkx346 28472356 PMC5570230

[39] R. Fraczkiewicz, W. Braun, Exact and efficient analytical calculation of the accessible surface areas and their gradients for macromolecules. J. Comput. Chem. 19, 319–333 (1998). 10.1002/(SICI)1096-987X(199802)19:3<319::AID-JCC6>3.0.CO;2-W

